# Shape-specific characterization of colorectal adenoma growth and transition to cancer with stochastic cell-based models

**DOI:** 10.1371/journal.pcbi.1010831

**Published:** 2023-01-23

**Authors:** Cristoforo Simonetto, Ulrich Mansmann, Jan Christian Kaiser

**Affiliations:** 1 Institute of Radiation Medicine, Helmholtz Munich, Oberschleissheim, Germany; 2 Institute for Medical Information Processing, Biometry, and Epidemiology, Ludwig-Maximilians-Universität, Munich, Germany; University of California Irvine, UNITED STATES

## Abstract

Colorectal adenoma are precursor lesions on the pathway to cancer. Their removal in screening colonoscopies has markedly reduced rates of cancer incidence and death. Generic models of adenoma growth and transition to cancer can guide the implementation of screening strategies. But adenoma shape has rarely featured as a relevant risk factor. Against this backdrop we aim to demonstrate that shape influences growth dynamics and cancer risk. Stochastic cell-based models are applied to a data set of 197,347 Bavarian outpatients who had colonoscopies from 2006-2009, 50,649 patients were reported with adenoma and 296 patients had cancer. For multi-stage clonal expansion (MSCE) models with up to three initiating stages parameters were estimated by fits to data sets of all shapes combined, and of sessile (70% of all adenoma), peduncular (17%) and flat (13%) adenoma separately for both sexes. Pertinent features of adenoma growth present themselves in contrast to previous assumptions. Stem cells with initial molecular changes residing in early adenoma predominantly multiply within two-dimensional structures such as crypts. For these cells mutation and division rates decrease with age. The absolute number of initiated cells in an adenoma of size 1 cm is small around 10^3^, related to all bulk cells they constitute a share of about 10^−5^. The notion of very few proliferating stem cells with age-decreasing division rates is supported by cell marker experiments. The probability for adenoma transiting to cancer increases with squared linear size and shows a shape dependence. Compared to peduncular and flat adenoma, it is twice as high for sessile adenoma of the same size. We present a simple mathematical expression for the hazard ratio of interval cancers which provides a mechanistic understanding of this important quality indicator. We conclude that adenoma shape deserves closer consideration in screening strategies and as risk factor for transition to cancer.

## Author summary

The incidence of colorectal cancer has been markedly reduced by removal of adenoma in screening colonoscopies. Adenoma contain precancerous cells which have already accumulated initial molecular changes on the pathway to cancer. Whereas adenoma size has long been recognized as a major cancer risk factor, the role of adenoma shape has not been studied intensively. Cell-based models ascribe the detected adenoma number and size in large screening cohorts to biological processes of early mutation, cell division, inactivation and transformation to cancer. They link different levels of biological organization which cannot be provided by mere statistical association. With biologically-based modeling we investigate shape-specific features of adenoma development. Model results reveal that adenoma of sessile, peduncular and flat shape exhibit differential growth dynamics, tendency of regression and carcinogenic potential.

## Introduction

Colorectal cancer ranks third in the list of worldwide cancer incidence and is the third leading cause of cancer death in Germany [1, 2]. Screening colonoscopies can effectively reduce the risk of contracting colorectal cancer by removal of adenoma as precursor lesions or timely detection of carcinoma [3]. Computer models are widely used to assess and devise screening strategies [4–6] and to predict the presence of high-risk adenoma [7] (and references therein). The conceptual design of these models is based on compartments which pertain to different phases of adenoma development on the pathway to cancer. For adenoma characterized with size and histology, transition rates between compartments are estimated by fits to large data sets from screening registries [6, 8].

Cell-based growth models are closer to biological reality and have been applied to assess the efficiency of different screening approaches or to predict colorectal cancer risk from adenoma detection [9, 10]. Jeon et al. [9] simulated adenoma prevalence with assumptions on size-dependent adenoma detection rates, but actual model fits were performed with cancer incidence data. Lang et al. [10] considered adenoma growth only for patients below age 50 yr and discarded screening data for older patients because they did not observe relevant adenoma increase in size. Both works [9, 10] represent stepping stones which leave room for a more detailed exploration of adenoma growth.

The majority of screening studies were not interested in adenoma shape as a risk factor which determines differential time patterns of growth and transition to cancer. Against this backdrop we hypothesize that consideration of adenoma shape has an impact on screening efficiency. We apply stochastic cell-based growth models and a simple model for transition to cancer to screening data of 197,437 Bavarian outpatients with colonoscopies in the years 2006–2009. We perform combined and separate investigations for sessile, peduncular and flat adenoma of both sexes. For the growth analysis we rely on the mathematical implementation of Dewanji et al. [11] for multi-stage clonal expansion (MSCE) models. The preferred growth models were identified by goodness-of-fit which measures their ability to simultaneously describe the distribution of adenoma number and size. Goodness-of-fit also decides whether clones of initiated cells in an adenoma prefer expansion in either two-dimensional (2d) or three-dimensional (3d) structures. With the preferred shape-specific models we predict the odds of pre-malignant cells in an adenoma to become either extinct or transit to cancer.

By descriptive evaluation of screening data Corley et al. [12] derived a hazard ratio for the risk of interval cancer which decreases with improved adenoma detection rate (ADR). We explain this much debated hazard ratio (see e.g. [13, 14]) by quantifying the dependence on adenoma number and size with a simple analytical expression. This expression highlights the capability of biologically-based modeling to provide mechanistic understanding for statistical associations.

Finally, we are investigating a data set out of clinical practice with sufficient detail to prevent the development of colorectal cancer by screening colonoscopy. It was not designed to meet our research aims and therefore has some limitations. Nevertheless, we strive to exploit the available information adequately with MSCE models to characterize the formation of colorectal adenoma and their transition to cancer. Wherever practicable we relate important model results to biological measurements and underline the clinical relevance.

## Materials and methods

### Bavarian outpatient colonoscopies

We analyze a subset of 258,116 records from outpatient colonoscopies which were performed by endoscopists as members of the Bavarian Association of Statutory Health Insurance Physicians (BASHIP or Kassenärztliche Vereinigung Bayerns) from January 2006 to December 2009 [15]. Colonoscopies were executed in symptom-free persons for prevention but not for diagnosis or surveillance. From multiple examinations of the same patient only the first examination was considered. 66,232 outpatients were diagnosed with adenoma or carcinoma. To study adenoma growth exclusively, we removed 471 records with carcinoma diagnosis. Compared to adenoma, carcinoma show a much faster growth and merit a separate treatment [16]. By demanding complete records for covariables histology, location, and adenoma size, count and location we arrived at 50,649 records with positive adenoma diagnosis. Age at screening ranged from 55 yr to 94 yr with mean age 65 yr similar for both sexes.

Adenoma counts were reported in three categories of 1, 2–4 or ≥ 5. In the count categories 2–4 and ≥ 5 the number of adenoma was set to 2 and 5, respectively. These are the most probable values under the assumption of Poisson-distributed counts. Under the same assumption the categorical means are estimated to about 2.1 and 5.2 from the recorded data. They came out similar for all shapes so that the bias from estimating counts in categories 2–4 and ≥ 5 can be neglected.

Linear size of adenoma has been grouped into four categories < 0.5, 0.5 − 1, 1 − 2 and > 2 cm. If more than one adenoma was detected only the category of the most advanced adenoma has been reported. Usually the most advanced adenoma acquired the largest size and we make this assumption in the present study. For the remaining adenoma (1 in count category 2–4 and 4 in count category ≥ 5) a size between the detection limit and the upper size bound of the largest adenoma must be assumed.

For the shape-specific analysis we assign the shape of the most advanced adenoma to all reported adenoma in count categories 2–4 and ≥ 5. Here we introduce a miss-classification bias which affects about 13% of patients with higher adenoma counts. We could have avoided miss-classification by analyzing the size distribution of the most advanced adenoma only. But in this case our models would overestimate the adenoma size. Since we are interested in a realistic size for cancer risk estimation we decided to keep miss-classification.

Records with negative adenoma diagnosis have been added to preserve the dependency of the adenoma detection rate (ADR) on age and sex [15]. The final data set for regression analysis comprised 197,347 records. In about 25% colonoscopies adenoma were detected with marked differences between women (20%) and men (32%).

In Fig A in S1 Text numbers of patients and ADRs for Bavarian patients are broken down in 5 yr age groups. Shape-specific numbers for sessile, flat and peduncular adenoma are given in Table B in S1 Text for both sexes. About 70% of adenoma were of sessile shape. The share of 18% peduncular adenoma was slightly higher compared to 12% flat adenoma. Fig B in S1 Text shows shape-specific patient numbers and ADRs.

Colorectal cancer cases have been recorded between 2006–2008 and are shown in Table C in S1 Text. By demanding completeness for the same covariables as for adenoma we arrived at 296 cases. Mean age at cancer was about 68 yr with little variation between shape and sex. In contrast to clinically relevant cases which prompted a diagnostic colonoscopy in hindsight only preclinical cases are considered in the present study.

### Adenoma growth model

#### Biological basics

The dominant molecular pathway in 70–80% of colon carcinoma is initiated by mutations in the APC gene [17, 18]. Initiated stem cells are susceptible to malignant transformation as opposed to cells that are differentiated and incapable of transforming. Stem cells do not belong to the bulk of glandular cells in an adenoma. They possess specific molecular changes which can in principle be characterized by adequate measurements. Inactivated APC genes cause an upregulation of the Wnt signaling pathway and enhancement of cell proliferation. APC mutations pertain to early adenoma. Further mutations in genes such as KRAS or DCC occur later in carcinogenesis. Only three driver mutations might be rate limiting [19]. These gene defects cause chromosomal instability (CIN) or aneuploidy which is associated with the loss of chromosomes 17p and 18. In late adenoma CIN cells have expanded into large clones before a transforming mutation often in the p53 gene triggers the development of invasive cancer.

In other pathways adenoma cells remain diploid with wild-type KRAS status. But they show more diverse molecular changes such as micro-satellite instability (MSI) and epigenetic silencing of the MLH1 gene from DNA methylation [17]. About 15–20% of colon carcinoma occur in these pathways and they are often collectively merged into a single MSI pathway.

#### Model concept

**Fig 1:**
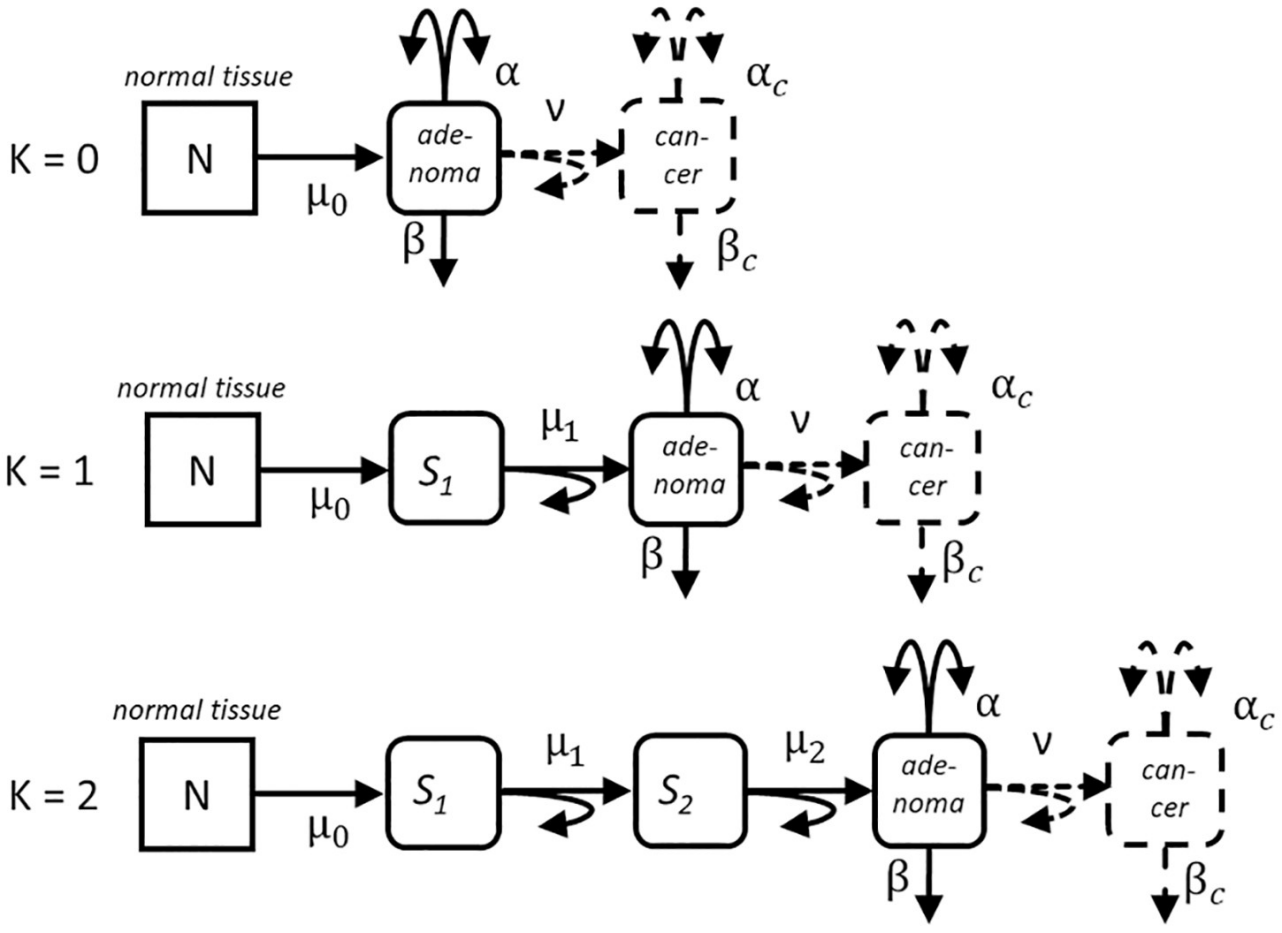
Three versions of the adenoma growth model. *N* normal stem cells of colonic crypts are susceptible to a series of mutations with rates *μ*_0_ (K = 0), *μ*_0_, *μ*_1_ with intermediate stage *S*_1_ (K = 1) and *μ*_0_, *μ*_1_, *μ*_2_ with intermediate stages *S*_1_, *S*_2_ (K = 2) before they start adenoma growth by clonal expansion with rates *α* for symmetric division and *β* for differentiation, *γ* = *α* − *β* is the net rate of clonal growth; dashes lines pertain to the full MSCE model of carcinogenesis (not modeled in the present study), *ν* denotes the transforming mutation rate to cancer cells, which expand into clinically relevant tumors with net rate *γ*_*c*_ = *α*_*c*_ − *β*_*c*_.

Colorectal adenoma are pre-neoplastic lesions which originate from *N* normal stem cells in colonic crypts [20]. Estimates for *N* range between 10^8^ − 10^9^ but do not influence model behavior [21, 22]. In at first healthy stem cells molecular changes are accumulated with different specification. The first step towards malignancy occurs for *Nμ*_0_ = *X*_*P*_ cells with rate *μ*_0_ where *X*_*P*_ is termed Poisson strength. Subsequent changes are represented as transition rates *μ*_*i*_ between cell stages *S*_*i*_ as shown in Fig 1. Conceptual models are distinguished by transition sequences of different length. The models are denoted as *K* = 0 with transition rate *μ*_0_, *K* = 1 with rates *μ*_0_, *μ*_1_ and *K* = 2 with rates *μ*_0_, *μ*_1_, *μ*_2_. Adenoma growth starts because cells with early molecular changes possess a growth advantage. The symmetric division rate *α* exceeds the inactivation rate *β* resulting in a net rate *γ* = *α* − *β* of clonal expansion. The last pre-clonal mutation rate *μ*_*K*_ is termed initiating, preceding mutations are considered as pre-initiating.

The implementation of the conceptual models is based on the mathematical framework and notation of Dewanji et al. [11, 23] and Jeon et al. [24] for the multi-stage clonal expansion (MSCE) model of cancer. By omission of the last transforming mutation *ν* and subsequent tumor growth with net rate *γ*_*c*_, the MSCE model is turned into a model of adenoma growth. The rate *ν* will be estimated separately with a simple model of transition to cancer. Parameters of conceptual models are derived from fits to shape-specific and combined adenoma data for both sexes separately. Following the approach of Kaiser et al. [25] for radiation-induced colorectal cancer, the preferred conceptual model is identified by goodness-of-fit.

#### Cell growth dimensionality

The growth model of Dewanji et al. [11] assumes that the number of adenoma and the number of initiated cells are known in principle for each patient. The adenoma number can be retrieved from the original data set (Table B in S1 Text). Measurements for the number of initiated cells per adenoma are not straightforward so that such numbers have to be estimated.

The stochastic growth model tracks probabilities for cell numbers per adenoma which accumulate with age, but is not concerned with the spatial distribution of cells. In the present study the unknown cell numbers are uniquely linked to adenoma size *S* measured in four categories < 0.5 cm, 0.5 − 1 cm, 1 − 2 cm and > 2 cm. Many studies assume that cell numbers increase with cubic power of linear size *S*^3^ [10, 24, 26, 27]. But early adenoma emerge from crypts as 2d structures. They are gradually converted into late adenoma and carcinoma with 3d volumetric forms. (see e.g. [17], their Fig 1). Therefore, we decided not to rely on the commonly accepted 3d cell heap model but to test both 2d and 3d growth patterns. The relation

$$\begin{array}{c} \frac{y}{y_{r}}=(\frac{S}{S_{r}}{)}^{d}\;\text{with}\;d=2,3 \end{array}$$ (1)

between cell number *y* and adenoma size *S* (measured in cm) does not assume a generic geometrical shape. Reference cell number *y*_*r*_ is related to reference size *S*_*r*_ = 1 cm. Goodness-of-fit will decide on predominantly 2d vs. 3d growth.

Reference cell numbers *y*_*r*_ have been assigned for different dimensions and shapes. For sessile and flat adenoma *y*_*r*_ is 800, 3200 in 2d, 3d respectively. For peduncular adenoma we assume *y*_*r*_ is 200 in 2d and 400 in 3d. The choices were informed by the observation that only the ratio *α*/*y*_*r*_ can be determined in model fits. Hence, we selected values for *y*_*r*_ so that estimates for cell division *α* remain in biologically plausible limits of about 5–50 yr^−1^ [22, 28, 29].

### Simple cancer risk model

The number of recorded cancer cases in Table C in S1 Text is far too low to support an extension of the adenoma growth model to a full MSCE model for cancer risk [16]. Hence, all three additional parameters *ν*, *α*_*c*_ and *γ*_*c*_ (see Fig 1) which describe the last steps of transformation and tumor growth on the pathway to cancer have not been estimated. In our simplified cancer risk model the transformation rate

$$\begin{array}{c} \nu \left(t\right)=\nu_{0}\;\text{exp}\;[(b_{n}\;\left(t-65\right)/10]. \end{array}$$ (2)

increases exponentially with age *t*. This behavior has been chosen because in the preferred adenoma growth models the number and size of adenoma does not increase strong enough to allow for a constant rate *ν*_0_. Since tumor growth is not considered explicitly, in effect *ν*(*t*) represents all biological processes which turn benign adenoma into tumors. *ν*(*t*) is linked to the adenoma growth model in Eq. (S46) and transforms the mean number of initiated cells per person to an epidemiological hazard rate.

### Regression analysis for adenoma growth models

#### Growth model selection

Model selection has been based on the three conceptual models for *K* = 0, *K* = 1 and *K* = 2 as depicted in Fig 1. For each conceptual model independent fits have been performed for the three shapes of adenoma combined and separately, for both sexes and for growth in two or three dimensions.

To identify the preferred models a systematic selection protocol has been executed on three levels. Level I started with estimation of three (*K* = 0: *X*_*P*_, *α*, *γ*) or four parameters (*K* = 1, 2: *X*_*PI*_, *α*, *γ*, *ρ*) without trends in attained age (Table 1). The detection limit was fixed to 0.25 cm for sessile and flat adenoma, and to 0.45 cm for peduncular adenoma. The specific choice for peduncular adenoma was made to accommodate the small number of 135 female and 191 male patients with peduncular adenoma of size < 0.5 cm. In level II the two parameters for the age trends of Eq (3) have been determined. Finally in level III the cell numbers which define detection limits have been optimized by trying a few selected values. Analyses for the Results section on comparison of measured and model-expected data and on straight model predictions have been performed with the preferred models of level III.

**Table 1:** Identifiable parameters for the growth models, models of level I apply parameters without trends in attained age, in models of levels II and III three additional parameters *b*_*x*_, $b_{x_{2}}$ and *b*_*a*_ pertain to age trends in Poisson strength *X*_*P*_, cell division *α*, net growth *γ* and initiation *ρ* according to Eq (3).

model version	parameter			
	Poisson strength/(pre-)initiation [yr^−1^]	^b^cell division [yr^−1^]	net growth [yr^−1^]	initiation [-]
*K* = 0	*X*_*PI*_ = *X*_*P*_	*α*	*γ* = *α* − *β*	-
*K* = 1	*X*_*PI*_ = *X*_*P*_	*α*	*γ* = *α* − *β*	*ρ* = *μ*_1_/*α*
^a^*K* = 2	^c^*X*_*PI*_ = *X*_*P*_*μ*_1_	*α*	*γ* = *α* − *β*	*ρ* = *μ*_2_/*α*

^a^ approximation *μ*_2_/*α* ≪ 1, Eq. (S34)

^b^ *α* estimate depends on reference cell number *y*_*r*_ at 1 cm

^c^ unit yr^−2^

In levels II and III parameter estimates for constant *α*_0_ (and *ρ*_0_ = *μ*_1,2_/*α*_0_ for *K* = 1, 2) have been fixed to stabilize the fitting process. Table D in S1 Text summarizes the flow of control for our regression analysis.

Since for analysis of ADR and adenoma per colonoscopy (APC) the shape covariable is of lesser clinical importance the results are derived for the model fitted to all shapes combined. The analysis of adenoma size and related cancer risk is presented separately for each shape. CIs from parameter uncertainties in growth models have been omitted for all calculated quantities because they came out generally small.

All analysis has been performed with packages of the R software suite [30]. For model fitting the R package bbmle [31] was selected because it allows for flexible parameter handling and for calculation of confidence intervals (CIs) from the likelihood profile.

#### Parameter identifiability and age trend

Not all biological parameters of the conceptual models from Fig 1 are identifiable by fits to the data [32]. Table 1 lists the selected set of identifiable parameters for the growth model. To include trends of attained age *a* the parameters *X*_*P*_ = *Nμ*_0_ for Poisson strength, cell division *α*, net growth *γ* and initiation *ρ* have been modified according to

$$\begin{array}{ccc} X_{P}\left(a\right) & = & X_{P0}\;\text{exp}\;(b_{x_{1}}a_{cen}+b_{x_{2}}a_{cen}^{2})\\ \alpha (a) & = & \alpha_{0}\;\text{exp}\;(b_{a}a_{cen})\\ \gamma (a) & = & \gamma_{0}\;\text{exp}\;(b_{a}a_{cen})\\ \rho (a) & = & \rho_{0}\;\text{exp}\;(-b_{a}a_{cen})\\ \;\text{with}\;a_{cen} & = & (a-65)/10. \end{array}$$ (3)

For fixed attained age the model parameters remain still constant. Since attained age ranges between 55–94 yr and data have been recorded in a short interval for calendar years 2006–09 the age dependences of Eq (3) also describe dependences on birth year in close approximation.

#### Individual likelihood and goodness-of-fit metrics

Dewanji et al. [11] developed likelihood functions for discrete cell numbers as cited in Eqs. (S36), (S38). The link between cell number and linear size is given by Eq (1). Because in the Bavarian data set *categories* for counts and sizes are given, the likelihood functions have been adjusted accordingly. The patient-specific likelihood function in Eq. (S40) is formed by two probabilities of 1) finding *n* adenoma with 2) sizes ranging between *y*_*h*_ and *y*_*l*_ cells. To allow for count categories Eq. (S40) has been extended to the likelihood functions of Eq. (S41).

Model parameters are estimated *simultaneously* for the probability distributions of adenoma counts and size. Adenoma counts are Poisson-distributed with the APC as mean value. Adenoma size depends on parameters *α*, *γ* (and *ρ* = *μ*_1,2_/*α* for *K* = 1, 2). Counts also depend on these parameters and *additionally* on Poisson strength *X*_*P*_.

To speed up the likelihood calculation in categories with more than one adenoma count the approximations of Eq. (S42) were applied. Individual records have been combined for each year of attained age, and for categories of adenoma size and number. Depending on shape the original sex-specific data sets of some 100,000 records shrunk to about 400–500 records without any loss of information. Statistical significance of model parameters were stated based on their p-values on the 95% level.

The deviance *D*_*I*_ for the individual likelihood is defined in Eq. (S43). It consists of two summands depending on either adenoma counts or size. Goodness-of-fit was measured by the Akaike Information Criterion *AIC* = *D*_*I*_ + 2*N*_*par*_ for *N*_*par*_ model parameters.

### Regression analysis for cancer risk models

To estimate the two parameters of the transformation rate *ν*(*t*) in Eq (2) the hazard of Eq. (S46)

$$\begin{array}{c} h\left(t\right)=\nu \left(t,\nu_{0},b_{n}\right)\;E\left[C\left(t,X_{PI},\alpha ,\gamma ,\rho \right)\right] \end{array}$$ (4)

was fitted to the crude rates of Table C in S1 Text by Poisson regression. Due to low case numbers only the parameters *ν*_0_ and *b*_*n*_ were estimated, but parameters *X*_*PI*_, *α*, *γ* and *ρ* of the growth model remained fixed.

Compared to CIs for quantities from the growth models, CIs for cancer hazards from the simple cancer risk models are much larger and are shown where appropriate. For the crude rates 95% CI have been derived by simulation based on the assumption of Poisson-distributed count statistics.

## Results

### Preferred models

For all three shapes combined and separately models with *K* = 0 were consistently outperformed in terms of *AIC* by models with one (*K* = 1) or two (*K* = 2) pre-initiating mutations in both levels I and II. In level I models with *K* = 1 with 2d growth showed the lowest *AIC*. In level II, where modifications for attained age of Eq (3) are applied to parameters, models with *K* = 2 took the lead. For flat adenoma 3d growth yields slightly better fits than 2d growth with *K* = 2 models. Details for goodness-of-fit are given in Tables E and F in S1 Text.

Level III models have been further optimized by adjusting the cell number of the detection limit. Goodness-of-fit and model parameters for the preferred level III models of 2d growth are given in Tables G—J in S1 Text. Table K in S1 Text pertains to a model for flat adenoma with 3d growth and *K* = 2. Given the small difference in goodness-of-fit with respect to growth dimensionality for flat adenoma, results for the model of 3d growth are occasionally compared to the preferred model of 2d growth.

### Parameter age trends

Parameter age trends are defined in Eq (3) for the growth models and in Eq (2) for the transformation rate of the cancer risk models. They are depicted in Figs C and D in S1 Text. Since screening data have been recorded in a short interval for calendar years 2006–09 age dependences cannot be disentangled from secular trends in birth cohort. Therefore, birth years for an assumed calendar year 2007 are indicated in the top x-axis of Figs C and D in S1 Text. In most cases the quadratic trend in Poisson strength *X*_*P*_ was statistically significant except for flat adenoma in women. For cell division rate *α* no significant quadratic trend was found (Tables G—K in S1 Text). For the transformation rate *ν* significant linear trends were found for all preferred models except for models of flat adenoma with 3d growth (Table L in S1 Text).

### Distribution of adenoma number

Results pertaining to APC, ADR and adenoma counts in categories have been derived from the model with *K* = 2 for all shapes combined. The latter quantities are used to measure screening efficiency and here adenoma shape is of lesser clinical relevance.

Fig E in S1 Text compares categorical adenoma counts for all shapes combined in 5 yr age groups with model expectations for women and men separately.

**Fig 2:**
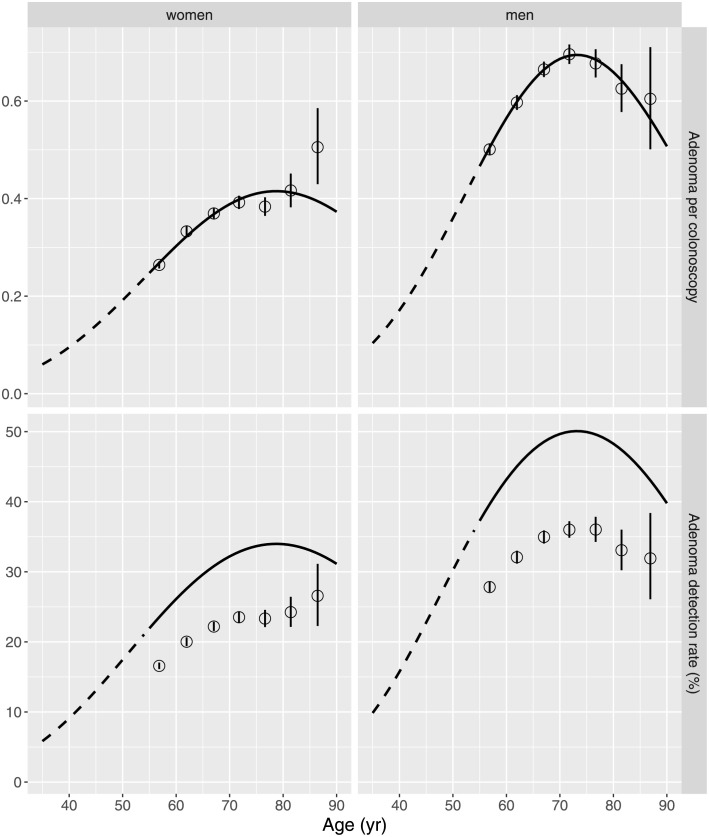
Measured mean number of adenoma per colonoscopy (APC) (top panels) and adenoma detection rate (ADR) (bottom panels) (open circles for 5 yr age groups with 95% CI) compared to the age dependence of APC and ADR (solid lines) expected by the preferred models for women (left panels) and men (right panels), dashed lines for age < 55 yr are model predictions.

Fig 2 displays a comparison between measured and model-expected APC and ADR, respectively. For Poisson-distributed adenoma counts APC constitutes an estimate of the Poisson mean.

### Distribution of adenoma size

Fig F in S1 Text compares measured patient shares with model expectations in size categories for all shapes combined. Table A in S1 Text summarizes the calculation of expected patient shares that was used to produce the data for Fig F in S1 Text. Since only the size of the largest adenoma is known, the share in the smallest size category depends on the ADR.

**Fig 3:**
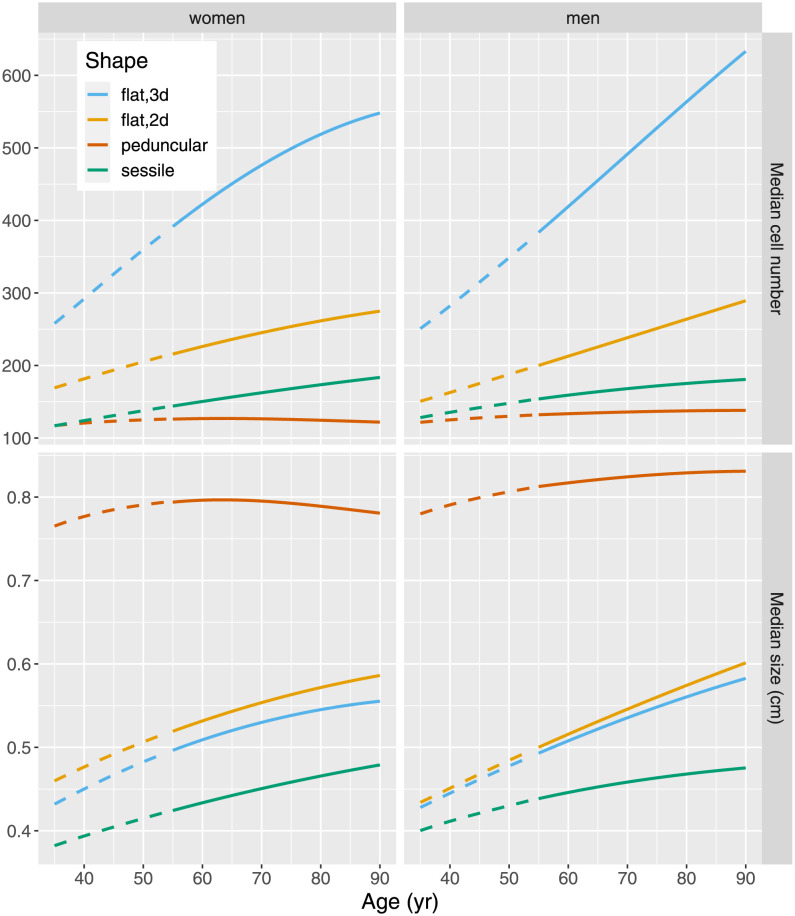
Median number of cells per adenoma (top panels) and median adenoma size in cm (bottom panels) for women (left panels) and men (right panels), dashed lines for age < 55 yr are model predictions.

Fig 3 depicts the shape-specific age dependence of the median cell number per adenoma (top panels) and median linear size (bottom panels).

#### Detection limit

For sessile and flat adenoma 30 initiated cells were identified as the optimal lower bound for the smallest size category of detectable adenoma. With Eq (1) this number corresponds to a detection limit of 0.19 cm for 2d growth or 0.21 cm in 3d. For peduncular adenoma 40 cells were needed with a detection limit of 0.45 cm in 2d.

#### Extinction probability

**Fig 4:**
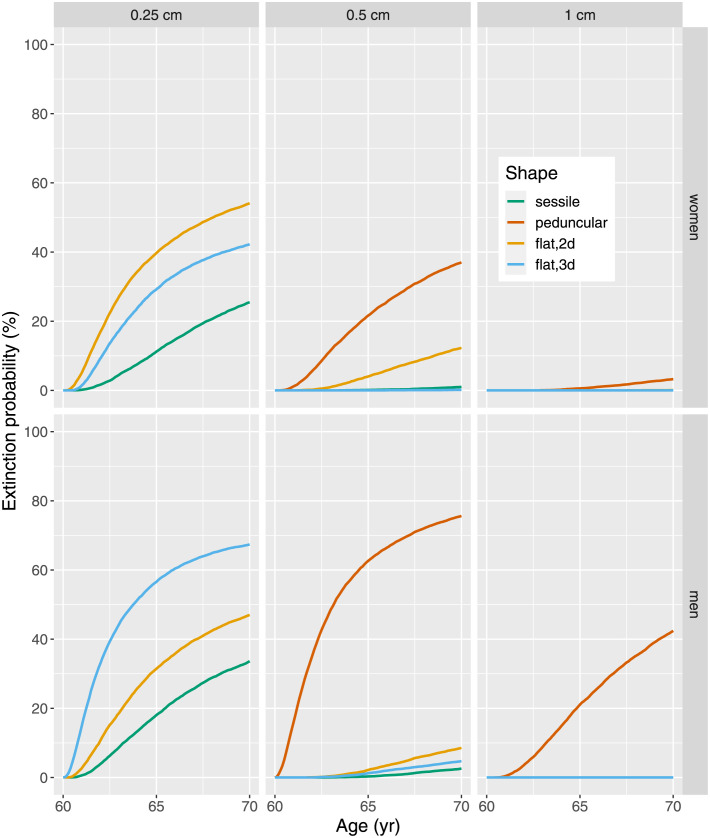
Shape-specific probability of extinction for clones of initiated cells present at age 60 yr for waiting time ≤ 10yr, clones correspond to adenoma with linear size 0.25, 0.5 and 1 cm for women (top panels) and men (bottom panels).

The extinction of clones with *y* initiated cells may occur with probability *P*_*ext*_(*y*, Δ*t*) defined in Eq. (S45). Fig 4 shows the age dependence of the extinction probability for 60 yr-old patients and a waiting time Δ*t* up to 10 yr.

### Cancer hazard

**Fig 5:**
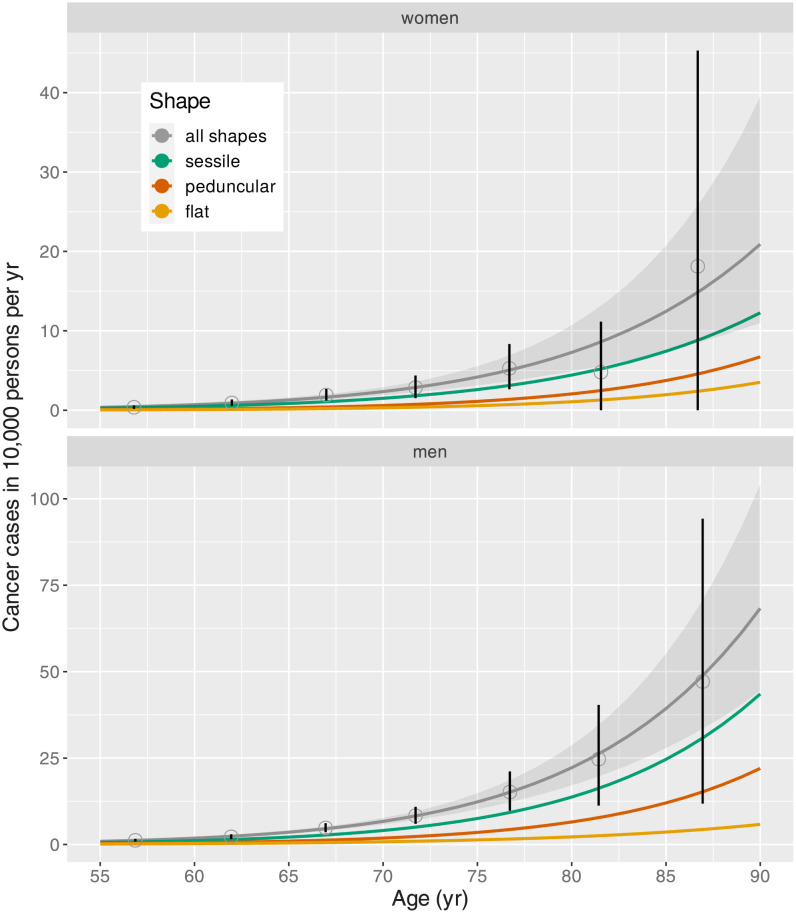
Age dependence of the shape-specific hazard from the simple cancer risk model, hazard for all shapes as sum of shape-specific functions (with shaded 95%CI) compared to crude rates for all shapes (point estimates with 95% CI) for women (top panel) and men (bottom panel).

Parameter estimates and 95% CI for the transformation rate (2) of the simple cancer risk model are given in Table L in S1 Text. The hazard functions of Eq (4) are visualized in Fig 5. The hazard for all shapes combined is given by the sum of shape-specific hazards which for flat adenoma included the hazard from the model with 3d growth.

### Probability of transition to cancer

**Fig 6:**
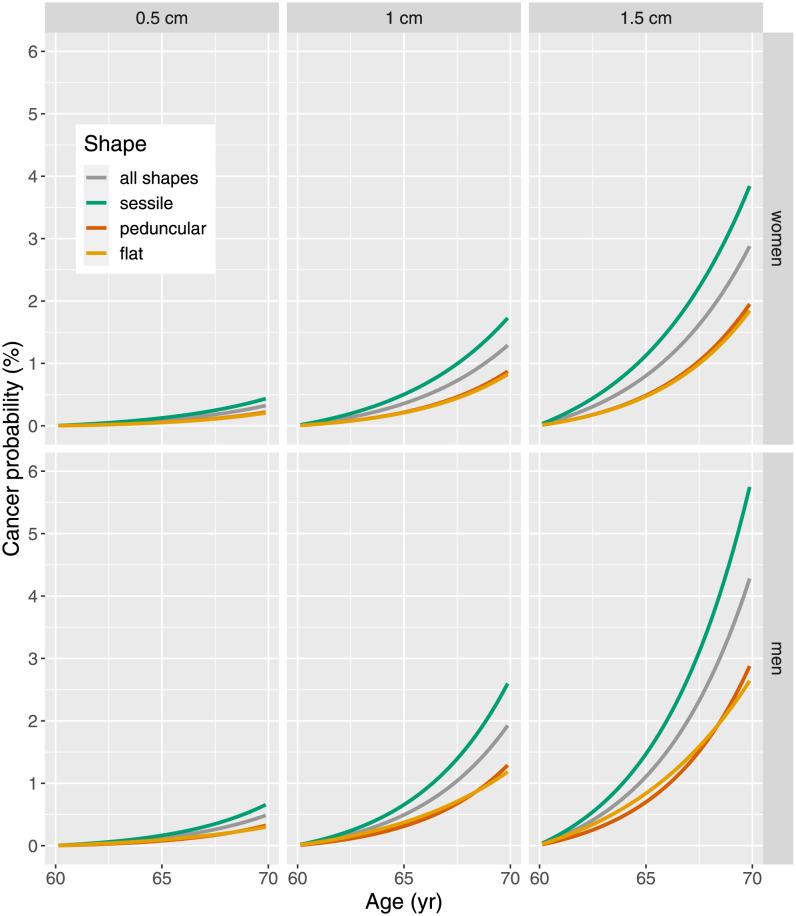
Shape-specific probability of transition to cancer for adenoma present at age 60 yr with size 0.5, 1 and 2 cm and waiting time up to 10 yr for women (top panels) and men (bottom panels).

Eq. (S49) defines the transition probability of an adenoma to cancer under the simplified assumption of an age-dependent transformation rate *ν*(*t*) (2). Estimates for this probability from Fig 6 do not take into account competing risks which are moderate between age 60 to 70 yr.

### Hazard ratios for risk of interval cancers

Interval cancers are diagnosed during time interval Δ*t* in patients after their colonoscopy at age *t*. In S1 Text we have derived a simple formula in Eq. (S58) for the relation between the hazard ratio HR_*eff*_(*t* + Δ*t*) of interval cancer and ADR which is repeated below as

$$\begin{array}{c} {\text{HR}}_{eff}≃(\frac{r_{N}^{eff}\;E_{N}^{y_{0}}\left(t\right)}{E_{N}^{0}\left(t\right)}-1)(\frac{r_{N}^{eff}\;E_{N}^{y_{0}}\left(t\right)}{E_{N}^{0}\left(t\right)}\frac{r_{Y}^{eff}\;E_{Y}^{y_{0}}\left(t\right)}{E_{Y}^{0}\left(t\right)}-1). \end{array}$$ (5)

The expression is valid under the assumption of negligible cancer risk from adenoma created *de novo* after a screening colonoscopy. In this case the risk for interval cancers is entirely related to incompletely removed or undetected adenoma.

The product $r_{N}^{eff}\;E_{N}^{y_{0}}\left(t\right)\equiv \text{APC(t)}$ defines APC with the effective removal factor $0<r_{N}^{eff}<1$ and the expected number of adenoma $E_{N}^{y_{0}}\left(t\right)$ at age *t* above the detection limit of *y*_0_ cells. If we assume that adenoma counts are Poisson distributed APC and ADR are monotonously related via APC(t) = −ln(1 − ADR(t)). An analogous definition with an effective reduction factor $0<r_{Y}^{eff}<1$ applies for expected size $E_{Y}^{y_{0}}\left(t\right)$ of detectable adenoma larger than *y*_0_.

**Fig 7:**
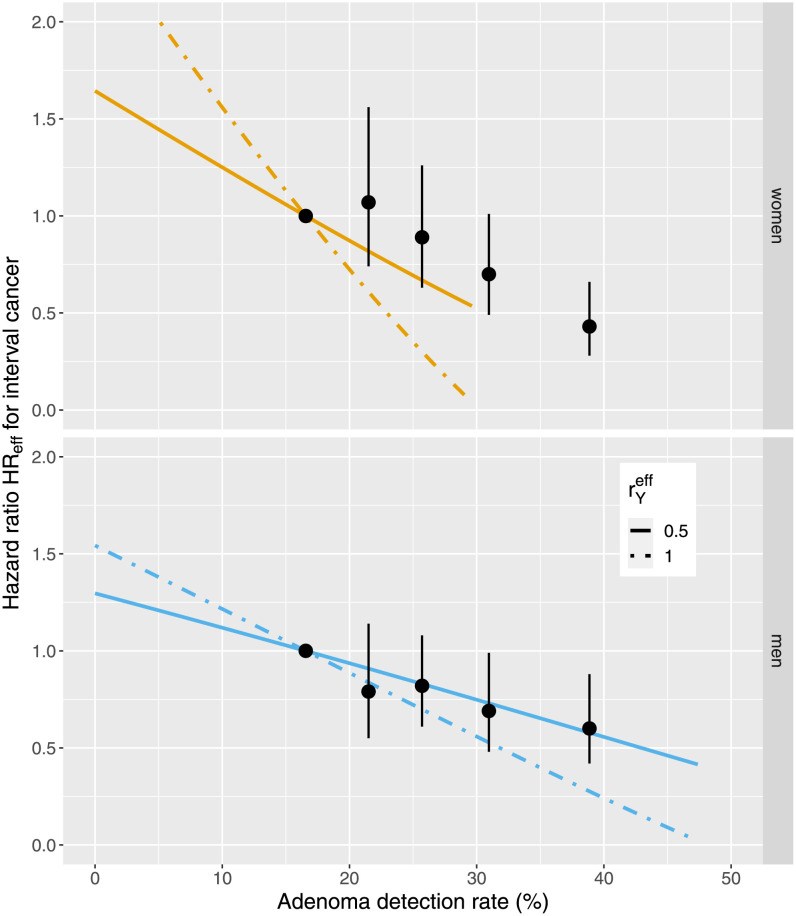
Comparison of hazard ratios *HR*_*eff*_ of Eq (5) for interval cancer depending on adenoma detection rate from the preferred model for all shapes combined for age at examination 65 yr (lines) and effective size reduction factor $r_{Y}^{eff}=1$ and 0.5, and from the study of Corley et al. [12] (point estimates with 95% CI) for women (top panel) and men (bottom panel).

Fig 7 compares model predictions for HR_*eff*_ from Eq (5) as a function of ADR from the preferred model for all shapes combined with estimates for the adjusted hazard ratio of Corley et al. [12] (their Supplementary Table S2). For the effective size reduction factor two values $r_{Y}^{eff}=0.5,1$ were assumed.

## Discussion

### Birth cohort and age trends

Shape-specific division rates *α* for tissue stem cells with initial molecular changes decelerate by about 20–40% between age 55–90 in Fig C in S1 Text. The effect was more pronounced for peduncular adenoma. Tomasetti et al. [33] have measured similar trends of around 40% reduction with Ki67 staining of normal cells obtained from human colonic crypts of patients in their 20s or 80s (see their Fig 2). Using Lgr5 as a marker for colonic *stem* cells in combination with Ki67 supported the trend but with much reduced counts of proliferating cells without statistical significance (see their Fig S5). Compared to the total number of evaluated cells the observed share of proliferating stem cells was in the order of 10^−5^.

Tomasetti and coworkers relate the deceleration in cell division rates to decreasing cancer incidence rates at old age. But they also point to the contribution of ‘weeding out of the susceptible’. We would interpret this contribution as a frailty effect which is an intrinsic feature of MSCE models [34]. However, the crude rates of preclinical cancer in the present study show no age-related attenuation in Fig 5. Due to the entanglement of secular trends with age trends in our screening cohort, comparison with age-dependent experimental data should be performed with caution.

On the other hand, we would not assign the increase in the transformation rate *ν* to a secular trend. A biologically plausible reason could involve malignant mutations (e.g. in gene p53) which accumulate with age and render a transition to cancer more likely.

### Adenoma number

Estimates of age-dependent APC reproduce the measurements closely in the top panels of Fig 2 but in the bottom panels the recorded ADR is strongly overestimated by model expectations. Fig E in S1 Text reveals a clear mismatch between model expectations and measurements in the categorical adenoma counts for all shapes combined. Similar results for shape-specific counts are not shown. Based on the assumption of a Poisson distribution the models expected more patients with exactly one adenoma and less patients without adenoma. In the Bavarian data set from 2006–2009 the ADRs are 20.4% for women and 30.5% for men almost equal to the ADRs reported by Brenner et al. [35] for whole Germany in 2013. In contrast the bottom panels of Fig 2 reveal higher model expectations of 30.0% for women and 48.0% for men at age 65 yr. For age 60 yr the models predict 26.4% for women and 40.7% for men. These values are in line with the results of Rutter et al. [36] for North American patients. They applied multinomial Poisson regression to a data set which combines information from several autopsy studies of adenoma prevalence and counts. The ADRs at age 60 yr in 1990 came out as 29.2% (women) and 40.3% (men).

Due to a compensation effect in the two lowest count categories APC estimates reflect the recorded data well. Given the good agreement of the model-expected ADR with more reliable autopsy studies [36] and the recorded ADR with other German data we attribute the ADR mismatch to overlooked adenoma. Fig F in S1 Text suggests that missing adenoma may mostly pertain to size < 0.5 cm. But categories for larger adenoma could also contribute. Therefore, our estimates of APC and ADR support a Poisson distribution. But they might come out slightly lower than biologically plausible estimates which are achievable for a given detection limit.

### Adenoma size

Size categories are reported only for the most advanced adenoma. Thus, a comparison of observed and expected size for all adenoma independent from count classification is not possible (Table A in S1 Text). Fig F in S1 Text shows undercounting of adenoma in lower size categories but cannot clarify the ability of the model to describe the size distribution adequately. A separate size analysis of the most advanced adenoma by multinomial Poisson regression could directly test the agreement of model expectations with measured data. However, such additional analysis would overestimate the actual adenoma size which is of clinical relevance in the present study.

Fig 3 reveals a strong shape-specific growth dynamics. Flat adenoma show a large positive age gradient whereas peduncular adenoma in women may even regress at older age. It is tempting to associate growth behavior with a shape-specific molecular profile but the published literature does not provide clear indications. The consensus molecular adenoma subtype classification does not consider shape as a relevant category [37]. Flat adenoma do not exhibit MSI, whereas in a few peduncular adenoma MSI characteristics have been reported [38–40]. Hallmarks of the MSI pathway are most likely to be found among serrated sessile adenoma but serration status was not available for our data set [41].

#### Number of precancerous cells per adenoma

By demanding biologically feasible division rates *α* between 5–50 *yr*^−1^, estimates for the number of initiated tissue stem cells per adenoma come out substantially lower compared to the range of 1–10% which has been reported previously [9, 26]. Based on a cell density of 10^8^ cm^−3^ [10] we obtain shares in the order of 10^−5^—10^−6^ initiated cells per adenoma. These small shares are based on absolute numbers at size 1 cm. Here we assume 800 cells for sessile adenoma with 2d growth, 200 cells for peduncular adenoma with 2d growth and 3200 cells for flat adenoma with 3d growth. Median cell numbers do not exceed 10^3^ for all shapes (Fig 3). In line with these findings, Tomasetti et al. [33] have measured a share of about 10^−5^ healthy stem cells which proliferate in the human colonic crypt.

Since the load of precancerous cells in an adenoma influences the risk of transition to cancer a determination of this quantity is of direct clinical relevance. Measurements of low cell numbers with distinct molecular signatures (see Komor et al. [37]) such as mutations in gene APC (CIN) or hypermethylation of gene MLH1 (MSI) can be performed by maximal depth sequencing, digital droplet PCR, and standard single cell RNA sequencing, which can detect altered copies in frequencies as low as 10^−5^ to 10^−6^ [42–44].

#### Adenoma regression

The extinction probability is considerable for small adenoma of all shapes, but with increasing size extinction becomes nearly impossible (Fig 4). Pickhardt et al. [27] performed a prospective trial to investigate the natural history of small adenoma < 1 cm. They measured *in vivo* growth rates with longitudinal CT colonography and report that 30% of small adenoma regressed with a volume reduction of 30%, and 10% completely resolved. They present tables with shares of adenoma which either progress, remain stable or regress. In principle, these tables can be used for model validation. However, for a comparison it must be kept in mind, that the number of bulk glandular cells in an adenoma exceeds the number of initiated stem cells with defined molecular changes by several orders of magnitude [33]. Hence, the extinction of an initiated clone does not necessarily imply the disappearance of the whole adenoma. To explain adenoma regression one must assume that cells with initial molecular changes take control over the fate of *all* cells in an adenoma. This assumption gains some support by Sievers et al. [45], who correlated the number of pathogenic mutations in small adenoma with their growth pattern. They show that small adenoma with just one pathogenic mutation are more likely to regress than adenoma with 2–3 mutations.

### Cancer risk

The shape-specific hierarchy in cancer hazard (Fig 5) is reflected in the cancer transition probabilities (Fig 6) for both sexes. Adenoma size is considered an important risk factor for transition, but this risk is markedly modified by adenoma shape. Sessile adenoma are on average smaller than flat and peduncular adenoma (Fig 3). Yet for the same size they exhibit a risk of transition larger by a factor of two. The large uncertainties in shape-specific crude rates strongly influence CIs for estimates of hazard functions. Transition probabilities (Fig 6) are burdened with similar uncertainties although they are not shown.

Our models predict transition probabilities proportional to size *S*^*d*^. For most adenoma this means a quadratic increase with linear size, but for flat adenoma a cubic increase is equally possible.

### Quality indicator for screening efficiency

Kaminski et al. [46] proposed the relation between the hazard ratio HR_*eff*_ for interval cancer and ADR as a quality indicator for screening efficiency. Corley et al. [12] have estimated that hazard ratio by statistical association for a large screening data set. Follow-up studies have emphasized the importance to increase ADRs for the reduction of colon cancer incidence and death [13, 14]). Motivated by the high clinical relevance we derived an analytic expression for HR_*eff*_ which is presented in Eq. (S58).

Age dependences of the ADR were very similar for outpatients from Bavaria and California with colonoscopies in overlapping periods of calendar years 2006 to 2008 [47]. Hence, comparison of hazard ratios for screening efficiency between Germany and the U.S. in Fig 7 allows us to assess the biological plausibility of the formula for HR_*eff*_ in Eq. (S58). Without adjustment of mean adenoma size ($r_{Y}^{eff}=1$) the preventive effect of screening is overestimated by our model. Good agreement between recorded data and modeling results for men is obtained by reducing mean adenoma size with $r_{Y}^{eff}=0.5$. This reduction might be associated with incomplete adenoma removal. For women borderline agreement is caused by the choice of the reference ADR for HR_*eff*_ = 1. The slight initial increase of the female HR_*eff*_ with increasing ADR in the Californian data is unexpected.

## Conclusion

We have presented a comprehensive characterization of adenoma growth and cancer transition risk with shape-specific analysis of screening colonoscopy data. Based on goodness-of-fit MSCE models with modifications tailored to meet the data format for Bavarian outpatients yielded important insights which are summarized below.

- Initiated cells in early adenoma of sessile and peduncular shape grow predominantly in 2d areal structures such as crypts. For flat adenoma this issue could not be resolved by goodness-of-fit and growth in 3d structures is equally possible.
- In line with measurements from Ki67 staining experiments cell division rates decelerate by about 20–40% for age 55–90 yr. Reduced proliferation might contribute to the attenuation of cancer incidence at old age.
- The number of stem cells with initial molecular damage is small in an adenoma. For linear size 1 cm we expect some 10^3^ initiated cells representing a share in the order of 10^−5^ related to all adenoma cells. These model expectations are supported by marker experiments and can be further validated by molecular measurements.
- Early adenoma grow markedly slower than colorectal tumors. The net clonal growth rates in 2d remain < 0.08 yr^−1^ whereas typical growth rates from fits to cancer incidence data are about twice as high.
- The risk for adenoma transiting to cancer shows a pronounced shape dependence. Compared to flat and peduncular adenoma the transition probability for sessile adenoma is about twice as high for the same linear size. For most adenoma transition probabilities increase with squared size.
- The hazard ratio for interval cancer serves as a screening quality criterion and has been previously quantified by statistical association. We now provide a mathematical expression based on a simple dependence on mean adenoma number and size which provides a mechanistic explanation.

Biologically-based modeling of adenoma growth and transition to cancer establishes a quantitative link between different levels of biological organization, which in this form cannot be provided by mere statistical association. Macroscopic measurements of adenoma size and number are explained by cell-based processes of molecular damage accumulation, cell division and clonal expansion. Depending on adenoma shape these processes proceed differently. Thus, we conclude that adenoma shape deserves closer consideration in screening strategies and as risk factor for transition to cancer.

## Supporting information

S1 Text Mathematical implementation and supplementary figures and tables. **Fig A**: Number of patients (A) and measured adenoma detection rate (B) in 5 yr age groups for all adenoma shapes combined. **Fig B**: Number of patients (A) and measured adenoma detection rate (B) in 5 yr age groups for shapes sessile, peduncular and flat; if more than one adenoma was reported, the shape of the most advanced adenoma was counted. **Fig C**: Age dependence of Poisson strength *X*_*P*_, cell division rate *α* and clonal growth rate *γ* defined in Eq (3), age dependence of initiation parameter *ρ*(*t*) = *μ*_2_/*α*(*t*) is not shown, parameter estimates and 95% CI are given in Tables G—K, parameter values are normalized to age 65, birth years on the top x-axis pertain to 2007—age. **Fig D**: Age dependence of the transformation rate *ν* defined in Eq (2), parameter estimates and 95% CI are given in Table L, birth years on the top x-axis pertain to 2007—age. **Fig E**: Adenoma counts of all shapes combined for men and women in 5 yr age groups from screening (left bars) and from model expectations (right bars), adenoma detection rate (ADR) is given by 1—Share(count = 0), *N*_*pat*_ denotes the number of patients in each panel. **Fig F**: Adenoma size distribution of all shapes combined for men and women in 5 yr age groups from screening (left bars) and from model expectations (right bars). If more than one adenoma is detected, the size category is derived from the size of the most advanced adenoma according to Table A, *N*_*pat*_ denotes the number of patients in each panel. **Table A**: Probabilities *P*(size = *sc*, counts = *cc*) of finding a patient with most advanced adenoma in size category *sc* and count category *cc*, for comparison with recorded adenoma the probabilities *P*(size = *sc*) = ∑_*cc*_ *P*(size = *sc*, counts = *cc*) in the last column have been used. **Table B**: Number of female and male patients *N*_*pat*_ and number of detected adenoma *N*_*ad*_ in 5 yr age groups. **Table C**: Number of female and male patients *N*_*pat*_, number of detected colorectal cancers *N*_*can*_ and crude incidence rate *cr* for 10^4^ persons per year in 5 yr age groups. **Table D**: Flow of control in regression analysis. **Table E**: Overall goodness-of-fit measured by *AIC* for all shapes combined, sex-specific models for *K* = 0, *K* = 1 and *K* = 2 with constant parameters (level I) and age dependences (3) in parameters *X*_*PI*_, *α* and *γ* (level II), cell growth was assumed in either 2d or 3d dimensions according to Eq (1). **Table F**: Overall goodness-of-fit measured by *AIC* shapes sessile, peduncular and flat separately, sex-specific models for *K* = 0, *K* = 1 and *K* = 2 with constant parameters (level I) and age dependences (3) in parameters *X*_*PI*_, *α* and *γ* (level II), cell growth was assumed in either 2d or 3d dimensions according to Eq (1). **Table G** Adenoma with all shapes combined: goodness-of-fit and parameter estimates for the preferred model *K* = 2 (level III), 2d growth and detection limit 30 cells (0.19 cm), age dependences in parameters *X*_*PI*_, *α* and *γ* from Eq (3). **Table H**: Sessile adenoma: goodness-of-fit and parameter estimates for the preferred model *K* = 2 (level III), 2d growth and detection limit 30 cells (0.19 cm), age dependences in parameters *X*_*PI*_, *α* and *γ* from Eq (3), p-values < 0.001. **Table I**: Peduncular adenoma: goodness-of-fit and parameter estimates for the preferred model *K* = 2 (level III), 2d growth and detection limit 40 cells (0.45 cm), age dependences in parameters *X*_*PI*_, *α* and *γ* from Eq (3), p-values < 0.001 unless indicated otherwise. **Table J**: Flat adenoma: goodness-of-fit and parameter estimates for the model *K* = 2 (level III), 2d growth and detection limit 30 cells (0.19 cm), age dependences in parameters *X*_*PI*_, *α* and *γ* from Eq (3), p-values < 0.001 unless indicated otherwise. **Table K**: Flat adenoma: goodness-of-fit and parameter estimates for the preferred model *K* = 2 (level III), 3d growth and detection limit 30 cells (0.21 cm), age dependences in parameters *X*_*PI*_, *α* and *γ* from Eq (3), p-values < 0.001 unless indicated otherwise. **Table L**: Parameter estimates for the transformation rate *ν*(*t*) = *ν*_0_ exp[(*b*_*n*_(*t* − 65)/10] in the simple cancer risk model, p-values < 0.001 unless indicated otherwise.
(PDF)
