# Supplementary material for: Shape-specific characterization of colorectal adenoma growth and transition to cancer with stochastic cell-based models
Source: PLoS Comput Biol. 2023 Jan 23;19(1):e1010831. doi: 10.1371/journal.pcbi.1010831 (PMC9894544; doi:10.1371/journal.pcbi.1010831)
Supplement: S1 Text — Fig A: Number of patients (A) and measured adenoma detection rate (B) in 5 yr age groups for all adenoma shapes combined. Fig B: Number of patients (A) and measured adenoma detection rate (B) in 5 yr age groups for shapes sessile, peduncular and flat; if more than one adenoma was reported, the shape of the most advanced adenoma was counted. Fig C: Age dependence of Poisson strength XP, cell division rate α and clonal growth rate γ defined in Eq (3), age dependence of initiation parameter ρ(t) = μ2/α(t) is not shown, parameter estimates and 95% CI are given in Tables G—K, parameter values are normalized to age 65, birth years on the top x-axis pertain to 2007—age. Fig D: Age dependence of the transformation rate ν defined in Eq (2), parameter estimates and 95% CI are given in Table L, birth years on the top x-axis pertain to 2007—age. Fig E: Adenoma counts of all shapes combined for men and women in 5 yr age groups from screening (left bars) and from model expectations (right bars), adenoma detection rate (ADR) is given by 1—Share(count = 0), Npat denotes the number of patients in each panel. Fig F: Adenoma size distribution of all shapes combined for men and women in 5 yr age groups from screening (left bars) and from model expectations (right bars). If more than one adenoma is detected, the size category is derived from the size of the most advanced adenoma according to Table A, Npat denotes the number of patients in each panel. Table A: Probabilities P(size = sc, counts = cc) of finding a patient with most advanced adenoma in size category sc and count category cc, for comparison with recorded adenoma the probabilities P(size = sc) = ∑cc P(size = sc, counts = cc) in the last column have been used. Table B: Number of female and male patients Npat and number of detected adenoma Nad in 5 yr age groups. Table C: Number of female and male patients Npat, number of detected colorectal cancers Ncan and crude incidence rate cr for 104 persons per year in 5 yr age groups. T [file pcbi.1010831.s001.pdf]

S1 Text

Shape-specific characterization of colorectal adenoma growth and transition to cancer with stochastic cell-based models

by Cristoforo Simonetto, Ulrich Mansmann, and Jan Christian Kaiser

last revised: January 17, 2023

S1 Mathematical implementation

Number and size distribution for $K = 0$

For $K = 0$ the unconditioned size distribution of pre-malignant clones created at age s with cell size $Y(s,t)$ at age t is given in Jeon et al. [1] (their Eqs. (3.31), (3.32))

$$\begin{aligned} p_y(s,t) &= P[Y(s,t) = y|\, Y(s,s) = 0] \\ &= \beta\zeta \quad \text{for} \quad y = 0 \\ &= (1-\beta\zeta)\,(1-\alpha\zeta)\,(\alpha\zeta)^{y-1} \quad \text{for} \quad y \geq 1 \\ \text{with} \quad \zeta(s,t) &= \frac{\exp\left[\gamma\,(t-s)\right]-1}{\alpha\,\exp\left[\gamma\,(t-s)\right]-\beta}. \end{aligned} \tag{S1}$$

Dewanji et al. [2] present an equivalent formulation in their Eq. (9)

$$\begin{aligned} p_y(s,t) &= P[Y(s,t) = y|\, Y(s,s) = 0] \\ &= 1 - \frac{1}{G(s,t)+g(s,t)} \quad \text{for} \quad y = 0 \\ &= \frac{1}{G(s,t)+g(s,t)}\,\frac{g(s,t)}{G(s,t)}\left(\frac{G(s,t)}{G(s,t)+g(s,t)}\right)^y \quad \text{for} \quad y \geq 1 \\ \text{with} \quad g(s,t) &= \exp\left[\gamma\,(s-t)\right], \\ G(s,t) &= \frac{\alpha}{\gamma}\,(1-g(s,t)) \\ \text{and} \quad \zeta(s,t) &= \frac{1}{\alpha}\,\frac{G(s,t)}{g(s,t)+G(s,t)}. \end{aligned} \tag{S2}$$

Adenoma can only be detected above a certain size which corresponds to a detection limit of y_0 cells. The number of detectable adenoma $N(t)$ is generated

by $M(t)$ initial mutations which occurred by age t at ages $s_i, i = 1, \ldots, M(t)$. Adenoma i may be detectable at age t ($N(s_i, t) = 1$ for $Y(s_i, t) > y_0$) or not ($N(s_i, t) = 0$ for $Y(s_i, t) \leq y_0$). Consequently,

$$N(t) = \sum_{i=1}^{M(t)} N(s_i, t) \tag{S3}$$

arises from a filtered Poisson process for the binary variable $N(s_i, t)$ with success probability

$$\begin{aligned} p^{(0)}(s,t) &= P[Y(s,t) > y_0 |\, Y(s,s) = 0] = 1 - \sum_{y=0}^{y_0} p_y(s,t) \\ &= \frac{1}{g(s,t) + G(s,t)} \left(\frac{G(s,t)}{G(s,t) + g(s,t)} \right)^{y_0} . \end{aligned} \tag{S4}$$

The summation has been carried out by exploiting properties of the partial geometric series. The integration over all possible time points s for the occurrence of a P_0-mutation yields Lerch's zeta function

$$\int_0^t \left(1 - \sum_{y=0}^{y_0} p_y(s,t) \right) \mathrm{d}s = \frac{1}{\alpha} \left[\alpha\zeta(t) \right]^{(1+y_0)} \; \mathrm{lerch}\left[\alpha\zeta(t), 1, 1 + y_0 \right] \tag{S5}$$

with $\zeta(t) = \zeta(0, t)$. The identity (S5) vanishes for $y_0 \to \infty$ and allows a fast computation of the quantities defined below for $K = 0$.

The expectation value for the number $N(t)$ of detectable adenoma is given by

$$E\left[N(t)\right] = \mu_0 X (\alpha\zeta)^{y_0} \zeta \; \mathrm{lerch}(\alpha\zeta, 1, 1 + y_0), \tag{S6}$$

and determines the probability

$$P\left[N(t)=0\right]=\exp\left(-E\left[N(t)\right]\right) \tag{S7}$$

of finding no detectable adenoma at age t.

For constant coefficients

$$P\left[Y(t)>y_0\right]=\frac{1}{t}(\alpha\zeta)^{y_0}\zeta\,\mathrm{lerch}(\alpha\zeta,1,1+y_0) \tag{S8}$$

denotes the probability of detecting an adenoma of size $Y(t)>y_0$ at age t.

As a special case the probability

$$\begin{aligned}P\left[Y(t)=0\right]&=1-\frac{1}{\alpha t}\ln\left(\frac{\alpha\exp(\gamma t)-\beta}{\gamma}\right)\\&\simeq\frac{\beta}{2}t+\frac{\beta}{6}(\alpha+\beta)t^2\quad\text{for}\quad t\to0\end{aligned} \tag{S9}$$

of finding no cell at age t is represented here in a compact expression.

The normalized size distribution of detectable adenoma at age t is given by

$$\begin{aligned}P[Y(t)=y|\,Y(t)>y_0]&=\frac{\Theta(y,t)}{E\left[N(t)\right]}\quad\text{with}\\\Theta(y,t)&=\mu_0X\int_0^t p_y(s,t)\mathrm{ds}\end{aligned} \tag{S10}$$

where for $K=0$ the expressions (S2), (S5) and (S6) apply.

Adenoma are measured in size intervals with lower and upper bounds which pertain to cell numbers $y_l<y_h$ above the detection limit y_0. The probability of finding an adenoma within these bounds

$$P[Y(t) \le y_h, Y(t) > y_l | \, Y(t) > y_0] = \sum_{y=y_l+1}^{y_h} P[Y(t) = y | \, Y(t) > y_0] \tag{S11}$$
$$= \frac{\Theta(y_l, y_h, t)}{E\,[N(t)]} \quad \text{with}$$
$$\Theta(y_l, y_h, t) = \frac{\mu_0 X}{\alpha} \left[(\alpha\zeta(t))^{1+y_l} \mathrm{lerch}(\alpha\zeta(t), 1, 1+y_l) \right.$$
$$\left. - \, (\alpha\zeta(t))^{1+y_h} \mathrm{lerch}(\alpha\zeta(t), 1, 1+y_h) \right]$$

can be calculated by applying identity (S5). If the upper cell size $y_h \to \infty$ the second term in $\Theta(y_l, y_h, t)$ pertaining to y_h disappears.

Based on the size distribution in Eq. (S10) cell-based expectation values such as the expected number of cells per detectable adenoma

$$E[Y(t)] = \frac{\int_0^t \sum_{y=y_0+1}^{\infty} y \, p_y(s,t) \mathrm{d}s}{(\alpha\zeta)^{y_0} \zeta \, \mathrm{lerch}(\alpha\zeta, 1, 1+y_0)} \tag{S12}$$
$$\text{with} \quad \sum_{y=y_0+1}^{\infty} y \, p_y(s,t) = \frac{1}{G(s,t)} \left(\frac{G(s,t)}{g(s,t) + G(s,t)} \right)^{y_0+1} \left(\frac{G(s,t)}{g(s,t)} + y_0 + 1 \right)$$

can be derived.

To compare with the measured mean adenoma size for d-dimensional growth the expectation value

$$E[Y^{1/d}(t)] = \frac{\int_0^t \sum_{y=y_0+1}^{\infty} y^{1/d} \, p_y(s,t) \mathrm{d}s}{(\alpha\zeta)^{y_0} \zeta \, \mathrm{lerch}(\alpha\zeta, 1, 1+y_0)} \tag{S13}$$

is needed. The size conversion factor

$$SC_d = \frac{S_r}{y_r^{1/d}} \tag{S14}$$

transforms the number initiated cells in an adenoma into physical adenoma size measured in cm. For $d = 2, 3$ y_r denotes the reference number of cells at reference size $S_r = 1$cm. The mean adenoma size is then given by $SC_d \times E[Y^{1/d}(t)]$.

Number and size distribution for $K = 1$ and $\mu_1/\alpha \ll 1$

By setting $\mu_1/\alpha \ll 1$ we assume that only a single sub-clone arises from P_0-mutated cells. Now we can draw on the equations for $K = 0$ and adjust them accordingly.

Starting from Eqs. (S4), (S6) the expectation value for the number $N(t)$ of detectable adenoma is given by

$$\begin{aligned} E\left[N(t)\right] &= \mu_0 X \int_0^t s\, p^{(0)}(s,t)\mathrm{d}s \qquad \text{(S15)} \\ &= \frac{\mu_0\mu_1 X}{\alpha}\alpha \int_0^t \frac{s}{g(s,t)+G(s,t)}\left(\frac{G(s,t)}{G(s,t)+g(s,t)}\right)^{y_0}\mathrm{d}s. \end{aligned}$$

The probability of detecting an adenoma of size $Y(t) > y_0$ and age t

$$\begin{aligned} P\left[Y(t) > y_0\right] &= \frac{1}{t}\int_0^t p^{(0)}(s,t)\mathrm{d}s \qquad \text{(S16)} \\ &= \frac{\mu_1}{\alpha}\frac{\alpha}{t}\int_0^t \frac{s}{g(s,t)+G(s,t)}\left(\frac{G(s,t)}{G(s,t)+g(s,t)}\right)^{y_0}\mathrm{d}s, \end{aligned}$$

and the normalized size distribution of detectable adenoma at age t

$$\begin{aligned} P[Y(t) = y|\, Y(t) > y_0] &= \frac{\int_0^t s\, p_y(s,t)\mathrm{d}s}{\int_0^t s\, p^{(0)}(s,t)\mathrm{d}s} \qquad \text{(S17)} \\ &= \frac{\Theta(y,t)}{E\left[N(t)\right]} \quad \text{with} \\ \Theta(y,t) &= \mu_0 X \int_0^t s\, p_y(s,t)\mathrm{d}s \end{aligned}$$

can be analogously calculated for $K = 1$ and $\mu_1/\alpha \ll 1$ from Eqs. (S4) and (S15).

Conveniently, we define the probability of finding no cell at age t

$$
\begin{aligned}
P\left[Y(t)=0\right] &= 1-\frac{\mu_1}{\alpha}\frac{\alpha}{t}\int_0^t \frac{s}{g(s,t)+G(s,t)}\mathrm{d}s \\
&= 1+\frac{\mu_1}{\alpha}\frac{\alpha}{t}\int_0^t \ln\left[1-\alpha\zeta(s,t)\right]\mathrm{d}s
\end{aligned}
\tag{S18}
$$

as a special case.

The probability of finding an adenoma within cell numbers y_l, y_h

$$
\begin{aligned}
P[Y(t)\le y_h, Y(t)>y_l|\,Y(t)>y_0] &= \sum_{y=y_l+1}^{y_h} P[Y(t)=y|\,Y(t)>y_0] \\
&= \frac{\Theta(y_l,y_h,t)}{E\left[N(t)\right]} \quad \text{with} \\
\Theta(y_l,y_h,t) &= \frac{\mu_0\mu_1 X}{\alpha}\alpha\left[\int_0^t \frac{s}{g(s,t)+G(s,t)}\left(\frac{G(s,t)}{G(s,t)+g(s,t)}\right)^{y_l}\mathrm{d}s\right. \\
&\quad \left.-\int_0^t \frac{s}{g(s,t)+G(s,t)}\left(\frac{G(s,t)}{G(s,t)+g(s,t)}\right)^{y_h}\mathrm{d}s\right]
\end{aligned}
\tag{S19}
$$

can be calculated by applying Eq. (S4).

Based on the size distribution in Eq. (S17) cell-based expectation values such as the expected number of cells per detectable adenoma

$$
E[Y(t)] = \frac{\int_0^t s\,\sum_{y=y_0+1}^{\infty} y\,p_y(s,t)\mathrm{d}s}{\int_0^t s\,p^{(0)}(s,t)\mathrm{d}s}
\tag{S20}
$$

$$
\text{with}\quad \sum_{y=y_0+1}^{\infty} y\,p_y(s,t) = \frac{1}{G(s,t)}\left(\frac{G(s,t)}{g(s,t)+G(s,t)}\right)^{y_0+1}\left(\frac{G(s,t)}{g(s,t)}+y_0+1\right)
$$

can be derived.

To compare with the measured mean adenoma size for d-dimensional growth the expectation value

$$E[Y^{1/d}(t)] = \frac{\int_0^t s \sum_{y=y_0+1}^{\infty} y^{1/d}\, p_y(s,t)\mathrm{d}s}{\int_0^t s\, p^{(0)}(s,t)\mathrm{d}s} \tag{S21}$$

is needed. By applying the size conversion factor from Eq. (S14) the mean adenoma size is then given by $SC_d \times E[Y^{1/d}(t)]$.

Number and size distribution for $K = 1$

After arrival of a P_1 cell with mutation rate μ_0 the unconditioned size distribution of a pre-malignant clone follows a Generalized Luria-Delbrück Distribution (GLD) [2]. For constant coefficients the GLD becomes a Negative Binomial Distribution [3]

$$\begin{aligned} p_y(s,t) &= P[Y(s,t) = y |\, Y(s,s) = 0] \\ &= \binom{\mu_1/\alpha + y - 1}{\mu_1/\alpha - 1} \left(1 - \alpha\zeta(s,t)\right)^{\mu_1/\alpha} \left(\alpha\zeta(s,t)\right)^y \\ \text{with} \quad \zeta(s,t) &= \frac{\exp\left[\gamma\left(t-s\right)\right] - 1}{\alpha \exp\left[\gamma\left(t-s\right)\right] - \beta}. \end{aligned} \tag{S22}$$

The probability $p_y(s,t)$ has the same meaning as in Eqs. (S1,S2) under the condition that a mutation leading to a P_1 cell has already occurred.

Below we repeat the formulation of the quantities for $K = 1$ related to Eqs. (S8) - (S12) for $K = 0$ as given in Dewanji et al. [3]

$$\begin{aligned} p^{(1)}(s,t) &= P[Y(s,t) > y_0 |\, Y(s,s) = 0] = 1 - \sum_{n=0}^{y_0} p_y(s,t) \\ &= \left(\alpha\zeta(s,t)\right)^{y_0+1} \left(1 - \alpha\zeta(s,t)\right)^{\mu_1/\alpha} \\ &\binom{\mu_1/\alpha + y_0}{\mu_1/\alpha - 1} {}_2F_1\left(1, 1 + \mu_1/\alpha + y_0, 2 + y_0, \alpha\zeta(s,t)\right) \\ &= p_{y_0+1}(s,t)\; {}_2F_1\left(1, 1 + \mu_1/\alpha + y_0, 2 + y_0, \alpha\zeta(s,t)\right) \end{aligned} \tag{S23}$$

using Eq. (S22) with the hypergeometric function ${}_2F_1$.

Analogously, the mathematical formulation for the expected number of adenoma

$$\begin{aligned} E\left[N(t)\right] &= \mu_0 X \int_0^t p^{(1)}(s,t)\mathrm{d}s \\ &= \mu_0 X \int_0^t p_{y_0+1}(s,t)\, {}_2F_1\left(1, 1+\mu_1/\alpha + y_0, 2+y_0, \alpha\zeta(s,t)\right)\mathrm{d}s, \end{aligned} \tag{S24}$$

the probability of detecting an adenoma of size $Y(t) > y_0$ at age t

$$\begin{aligned} P\left[Y(t) > y_0\right] &= \frac{1}{t}\int_0^t p^{(1)}(s,t)\mathrm{d}s \\ &= \frac{1}{t}\int_0^t p_{y_0+1}(s,t)\, {}_2F_1\left(1, 1+\mu_1/\alpha + y_0, 2+y_0, \alpha\zeta(s,t)\right)\mathrm{d}s, \end{aligned} \tag{S25}$$

and the normalized size distribution of detectable adenoma at age t

$$\begin{aligned} P[Y(t) = y|\, Y(t) > y_0] &= \\ &\frac{\int_0^t p_y(s,t)\mathrm{d}s}{\int_0^t p_{y_0+1}(s,t)\, {}_2F_1\left(1, 1+\mu_1/\alpha + y_0, 2+y_0, \alpha\zeta(s,t)\right)\mathrm{d}s} \\ &= \frac{\Theta(y,t)}{E\left[N(t)\right]} \quad \text{with} \\ \Theta(y,t) &= \mu_0 X \int_0^t p_y(s,t)\mathrm{d}s \end{aligned} \tag{S26}$$

can be constructed for $K = 1$ from the expressions (S22), (S23) and (S24).

Additionally, we present

$$P\left[Y(t) = 0\right] = \frac{1}{t}\int_0^t \left[1 - \alpha\zeta(t,s)\right]^{\mu_1/\alpha}\mathrm{d}s \tag{S27}$$

as the probability of finding no cell at age t.

From Eq. (S26) follows probability of finding an adenoma in interval $(y_l, y_h]$

$$P[Y(t) \leq y_h, Y(t) > y_l | \, Y(t) > y_0] = \sum_{y=y_l+1}^{y_h} P[Y(t) = y | \, Y(t) > y_0] \tag{S28}$$
$$= \frac{\Theta(y_l, y_h, t)}{E\,[N(t)]} \quad \text{with}$$
$$\Theta(y_l, y_h, t) = \mu_0 X \left[\int_0^t p_{y_l+1}(s,t) \; {}_2F_1\left(1, 1+\mu_1/\alpha + y_l, 2 + y_l, \alpha\zeta(s,t)\right) \mathrm{d}s \right.$$
$$\left. - \int_0^t p_{y_h+1}(s,t) \; {}_2F_1\left(1, 1+\mu_1/\alpha + y_h, 2 + y_h, \alpha\zeta(s,t)\right) \mathrm{d}s \right].$$

for $K = 1$. If the upper cell size $y_h \to \infty$ the second term in $\Theta(y_l, y_h, t)$ pertaining to y_h disappears.

Based on the size distribution for $K = 1$ in Eq. (S26)

the values

$$E[Y(t)] = \tag{S29}$$
$$\frac{\int_0^t \sum_{y=y_0+1}^{\infty} y \, p_y(s,t) \mathrm{d}s}{\int_0^t p_{y_0+1}(s,t) \; {}_2F_1\left(1, 1+\mu_1/\alpha + y_0, 2 + y_0, \alpha\zeta(s,t)\right) \mathrm{d}s}$$

and

$$E[Y^{1/d}(t)] = \frac{\int_0^t \sum_{y=y_0+1}^{\infty} y^{1/d} \, p_y(s,t) \mathrm{d}s}{\int_0^t p_{y_0+1}(s,t) \; {}_2F_1\left(1, 1+\mu_1/\alpha + y_0, 2 + y_0, \alpha\zeta(s,t)\right) \mathrm{d}s}$$

for the expected cell number $E[Y(t)]$ and $E[Y^{1/d}(t)]$ at age t must be calculated numerically. Multiplication of the size conversion factor SC_d of Eq. (S14) to $E[Y^{1/d}(t)]$ yields the mean adenoma size in cm from growth in $d = 2, 3$ dimensions, respectively.

Number and size distribution for $K = 2$ and $\mu_2/\alpha \ll 1$

For constant model parameters the expected number of adenoma is given by

$$\begin{aligned} E\left[N(t)\right] &= \mu_0\mu_1 X \int_0^t \int_s^t p^{(1)}(s',t)\mathrm{d}s'\mathrm{d}s \\ &= \mu_0\mu_1 X \int_0^t s\, p_{y_0+1}(s,t)\ {}_2F_1\left(1, 1+\mu_2/\alpha+y_0, 2+y_0, \alpha\zeta(s,t)\right)\mathrm{d}s, \end{aligned} \tag{S30}$$

using the probability of success $p^{(1)}(s,t)$ defined in Eq. (S23) (Dewanji et al. [3] (their Eq. (22)).

The detection probability [3] (their Eq. (23))

$$\begin{aligned} P\left[Y(t) > y_0\right] &= \frac{2}{t^2}\int_0^t s\, p^{(1)}(s,t)\mathrm{d}s \\ &= \frac{2}{t^2}\int_0^t s\, p_{y_0+1}(s,t)\ {}_2F_1\left(1, 1+\mu_2/\alpha+y_0, 2+y_0, \alpha\zeta(s,t)\right)\mathrm{d}s, \end{aligned} \tag{S31}$$

and the normalized size distribution of detectable adenoma at age t [3] (their Eq. (24))

$$\begin{aligned} &P[Y(t) = y|\, Y(t) > y_0] = \\ &\qquad \frac{\int_0^t s\, p_y(s,t)\mathrm{d}s}{\int_0^t s\, p_{y_0+1}(s,t)\ {}_2F_1\left(1, 1+\mu_2/\alpha+y_0, 2+y_0, \alpha\zeta(s,t)\right)\mathrm{d}s} \\ &\qquad = \frac{\Theta(y,t)}{E\left[N(t)\right]} \quad \text{with} \\ &\Theta(y,t) = \mu_0\mu_2 X \int_0^t s\, p_y(s,t)\mathrm{d}s \end{aligned} \tag{S32}$$

can be constructed for $K = 2$ in the same way for as for $K = 1$ by obeying $\int_0^t \int_s^t p(s',t)\mathrm{d}s' = \int_0^t s\, p(s,t)\mathrm{d}s$.

As a special case of Eq. (S31) it is convenient to present

$$P\left[Y(t)=0\right]=\frac{2}{t^2}\int_0^t s\,\left[1-\alpha\zeta(t,s)\right]^{\mu_2/\alpha}\mathrm{d}s \tag{S33}$$

as the probability of finding no cell at age t,

From Eq. (S32) follows the probability of finding an adenoma in the interval $(y_l, y_h]$

$$\begin{aligned}
P[Y(t)\le y_h, Y(t)>y_l|\,Y(t)>y_0] &= \sum_{y=y_l+1}^{y_h} P[Y(t)=y|\,Y(t)>y_0] \qquad \text{(S34)}\\
&= \frac{\Theta(y_l,y_h,t)}{E\left[N(t)\right]} \quad \text{with}\\
\Theta(y_l,y_h,t) &= \mu_0\mu_1 X\left[\int_0^t s\,p_{y_l+1}(s,t)\;{}_2F_1\left(1,1+\mu_2/\alpha+y_l,2+y_l,\alpha\zeta(s,t)\right)\mathrm{d}s\right.\\
&\quad\left.-\int_0^t s\,p_{y_h+1}(s,t)\;{}_2F_1\left(1,1+\mu_2/\alpha+y_h,2+y_h,\alpha\zeta(s,t)\right)\mathrm{d}s\right].
\end{aligned}$$

If the upper cell size $y_h\to\infty$ the second term in $\Theta(y_l,y_h,t)$ pertaining to y_h disappears.

For $K=2$ the expected cell number $E[Y(t)]$ and $E[Y^{1/d}(t)]$ at age t are defined analogously as for $K=1$ in Eq. (S29)

$$E[Y(t)] = \tag{S35}$$

$$\frac{\int_0^t s\,\sum_{y=y_0+1}^{\infty} y\,p_y(s,t)\mathrm{d}s}{\int_0^t s\,p_{y_0+1}(s,t)\;{}_2F_1\left(1,1+\mu_2/\alpha+y_0,2+y_0,\alpha\zeta(s,t)\right)\mathrm{d}s}$$

and

$$E[Y^{1/d}(t)] = \frac{\int_0^t s\,\sum_{y=y_0+1}^{\infty} y^{1/d}\,p_y(s,t)\mathrm{d}s}{\int_0^t s\,p_{y_0+1}(s,t)\;{}_2F_1\left(1,1+\mu_2/\alpha+y_0,2+y_0,\alpha\zeta(s,t)\right)\mathrm{d}s}.$$

Dewanji et al. [3] define a 'special' P_1-mutation as the origin of at least one detectable adenoma. For constructing the likelihood function for $K = 2$ we assume that from each 'special' P_1-mutation just one (and not several) adenoma arises. This is the case if $\mu_1 \int_s^t p^{(1)}(s', t)\mathrm{d}s' < \mu_1\, t \ll 1$. Hence, the probability of harboring a 'special' P_1-mutation during lifetime must be small. In this case the joint probability of having n detectable adenoma [3] (their Eq. (26)) reduces to Eq. (S32). This approximation leads to a substantial simplification of the likelihood function [3] (their Eq. (27)) which for $K = 2$ can now be couched in the same general form developed below.

Likelihood function for adenoma with discrete cell numbers

The contribution $\mathcal{L}_j$ of patient j to the total likelihood $\mathcal{L} = \prod_j^{N_{pat}} \mathcal{L}_j$ for N_{pat} patients is given by

$$\mathcal{L}_j = P\left[N(t_j) = n\right] \prod_{i=1}^{n} P\left[Y(t_j) = y_i \mid Y(t_j) > y_0\right]. \tag{S36}$$

if n adenoma of size $y_i, i = 1 \ldots n$ have been detected at age t_j with a detection limit of y_0 cells [3].

In Eq. (S36) $P\left[N(t_j) = n\right]$ denotes the probability of detecting n adenoma above the detection limit and $P\left[Y(t_j) = y_i \mid Y(t_j) > y_0\right]$ denotes the probability that each of these adenoma consists of $y_i > y_0$ cells.

The number of adenoma $N(t_j)$ has a Poisson distribution with mean $E[N(t_j)]$ which is determined by the choice of the growth model. For

$$P\left[N(t_j) = n\right] = \frac{E[N(t_j)]^n}{n!} \exp(-E[N(t_j)]) \tag{S37}$$

$$P\left[Y(t_j) = y_i \mid Y(t_j) > y_0\right] = \Theta(y_i, t_j)/E[N(t_j)]$$

one gets

$$\mathcal{L}_j \sim \exp\left(-E[N(t_j)]\right) \prod_{i=1}^{n} \Theta(y_i, t_j) \tag{S38}$$

with the model-specific expectation value $E[N(t_j)]$ for the number of detectable adenoma from Eqs. (S6), (S24) and (S30). The model-specific function $\Theta(y_i, t_j)$ from Eqs. (S10), (S26) and (S32) is determined by the number of cells y_i in adenoma i at age t_j of patient j.

Likelihood function for adenoma in categories of size and number

To meet the layout of the data set for Bavarian outpatients the likelihood function of Eq. (S36) [3] needs to be modified. The Bavarian data set reports the prevalence of adenoma in patient j in size categories and categories for the number of adenoma. If several adenoma were detected only the size category of the most advanced adenoma is indicated. Here we assume that the most advanced status implies the largest adenoma size. For this size category y_l, y_h denote the cell numbers of the lower and upper boundary, respectively. For the remaining adenoma we must assume a size between the detection limit at y_0 and the upper bound of the largest adenoma y_h.

The definitions

$$P_l(t) = P[Y(t) \leq y_l, Y(t) > y_0 | \, Y(t) > y_0] \tag{S39}$$

$$P_h(t) = P[Y(t) \leq y_h, Y(t) > y_0 | \, Y(t) > y_0]$$

for models $K = 0$ in Eq. (S11), $K = 1$ in Eq. (S28) and $K = 2$ in Eq. (S34) are helpful to present the likelihood function.

The resulting probability for the size distribution of n adenoma is based on $\frac{n!}{k!(n-k)!}$ possible permutations for k indistinguishable adenoma in the size category of the largest adenoma. The condition $n \geq k \geq 1$ ensures that at least one adenoma populates this category. Hence, with Eq. (S37) the contribution $\mathcal{L}_j$ of patient j to the total likelihood function becomes

$$\mathcal{L}_j = P\left[N(t_j) = n\right]\left[P_h(t_j)^n - P_l(t_j)^n\right] \tag{S40}$$

analogous to Eq. (S38).

In the Bavarian data set four categories for the number n of detected adenoma are provided: $n \in \{0\}$ with detection limit y_0, $n \in \{1\}$, $n \in \{2,3,4\}$ and $n \in \{5,\ldots,\infty\}$. Following Eq. (S39) we introduce the cumulative probability $\Theta = \Theta(y_0, y_h, t)$ for cell numbers between y_0 and the upper boundary y_h, and the corresponding cumulative probability $\vartheta = \Theta(y_0, y_l, t)$ for the lower boundary y_l. By starting from the patient-specific likelihood (S40) with $E[N(t)] = \lambda$ we obtain

$$\begin{aligned}
\mathcal{L}_j &= e^{-\lambda} \quad \text{for} \quad n \in \{0\}, \qquad \text{(S41)}\\
\mathcal{L}_j &= e^{-\lambda}\,\Theta \quad \text{for} \quad n \in \{1\},\\
\mathcal{L}_j &= e^{-\lambda} \sum_{n=2}^{4} \frac{\Theta^n - \vartheta^n}{n!} \quad \text{for} \quad n \in \{2,3,4\},\\
\mathcal{L}_j &= e^{-\lambda} \left(e^{\Theta} - e^{\vartheta} - \sum_{n=0}^{4} \frac{\Theta^n - \vartheta^n}{n!} \right) \quad \text{for} \quad n \in \{5,\ldots,\infty\}.
\end{aligned}$$

The model-specific Θ-functions are defined in Eqs. (S11), (S28) and (S34). Note, that $\Theta = \lambda$ for $y_h \to \infty$ in the largest size category and $\vartheta = 0$ for $y_l = y_0$ in the smallest size category.

To simplify Eq. (S41) we set the number of adenoma to 2 for $n \in \{2,3,4\}$ and to 5 for $n \in \{5,\ldots,\infty\}$. These numbers pertain to maximal Poisson probabilities per count category. For patients with more than one adenoma we obtain

$$\mathcal{L}_j \simeq e^{-\lambda} \frac{\Theta^2 - \vartheta^2}{2} \sim e^{-\lambda} \left(\Theta^2 - \vartheta^2\right) \quad \text{for} \quad n =\in \{2, 3, 4\}, \tag{S42}$$
$$\mathcal{L}_j \simeq e^{-\lambda} \frac{\Theta^5 - \vartheta^5}{120} \sim e^{-\lambda} \left(\Theta^5 - \vartheta^5\right) \quad \text{for} \quad n \in \{5, \ldots, \infty\}.$$

Estimates of identifiable model parameters are obtained from the deviance

$$D_I = -2 \ln \hat{\mathcal{L}} \tag{S43}$$

defined with the maximized likelihood function $\hat{\mathcal{L}} = \prod_j \hat{\mathcal{L}}_j$.

Conditional size distribution

If more than one adenoma has been detected in a patient, only properties (i.e size or histology) of the most advanced adenoma has been reported. This lack of information in the data set does not allow a direct comparison of recorded and model-expected size distributions for *all* adenoma. The size distribution can only be estimated conditioned on the count category, for which the adenoma size has been reported. A breakdown of the probabilities $P(\text{size} = sc, \text{counts} = cc)$ is given in Table A below for size categories $sc =$ $<$ 0.5 cm with probability p_1, 0.5 - 1 cm with probability p_2, 1 - 2 cm with probability p_3 and $\geq$ 2 cm with probability p_4. The probabilities p_i add up to one and are given in the model-specific Eqs. (S11), (S28) and (S34).

The counts are Poisson distributed according to $d_n = \frac{\lambda^n}{n!} e^{-\lambda}$ in categories $cc =$ 0, 1, 2-4 and $\geq$ 5. The expectation value λ for the number of adenoma is given in the model-specific Eqs. (S6), (S24) and (S30). The relation

$$\sum_{n=0}^{\infty} d_n p_i^n = e^{\lambda(p_i - 1)} \tag{S44}$$

has been applied to calculate the sum over count categories in Table A.

Extinction probability

The conditional probability for a clone with y initiated cells at age t_b to become extinct at age t_e is

$$P[Y(t_e) = 0|Y(t_b) = y] = P[Y(t_b) = y]\, P_{ext}(y, t_e - t_b). \qquad \text{(S45)}$$

For constant parameters the extinction probability $P_{ext}(y, t_e - t_b)$ depends on the number of initiated cells y and waiting time Δt. We have derived this probability from simulation of 10,000 clones which have been propagated in time steps of $\Delta t = 0.1$ yr. To update the clone size we assume Poisson distributions for $\mu_k \Delta t$ new cells arriving from P_K-mutations, $\alpha \Delta t$ new cells from cell division and $\beta \Delta t$ vanishing cells due to inactivation. A clone is considered extinct if the update leads to a cell number ≤ 0. The extinction probability is then given by the ratio of the number of extinct clones over the total number of simulated clones. We do not consider transition to cancer with Poisson mean $\nu(t)\Delta t$ which is usually very small (Table L). Since the contribution from newly initiated cells via P_K-mutations is also quite small, the extinction probability is mainly determined by the relation between α and β. Note, that the extinction probability pertains to clones of initiated cells but *not* to whole adenoma which comprise a by orders of magnitude higher number of cells.

Cancer risk

Hazard functions

In the MSCE framework the hazard $h(t)$ at age t for incidence of colorectal cancer increases linearly with transition rate ν which transforms a single cell from an initiated adenoma into full-blown cancer. The hazard is given by

$$\begin{aligned} h(t) &= \nu\, E[N(t - t_{lag})|y > 0] E[Y(t - t_{lag})|y > 0] \\ &= \nu\, E[C(t - t_{lag})|y > 0], \end{aligned} \tag{S46}$$

where $E[C(t - t_{lag})|y > 0]$ denotes the mean number of initiated cells per individual which are susceptible to transformation into cancer. Tumor growth can be described in the same way as adenoma growth with a net expansion rate γ_C (see Fig. 1). By simplification γ_C is replaced with a lag time t_{lag} which must elapse before a tumor becomes clinically relevant. For colorectal cancer Luebeck et al. [4] give estimates of about 2 yr^{-1} for γ_C, 5 yr for t_{lag} and 10^{-6} yr^{-1} for ν. For the growth rate γ of precancerous clones they estimate 0.15 yr^{-1} for women and 0.16 yr^{-1} for men. In parameter estimation for the simple cancer risk model t_{lag} is neglected.

For small ν (i.e. $< 10^{-5}$ yr^{-1}) the expectation values for the number of non-extinct adenoma $E[N(t)|y > 0]$ are given for each model in Eqs. (S6) ($K = 0$), (S24) ($K = 1$) and (S30) ($K = 2$). The corresponding expectation values for the number of cells per adenoma are found in Eqs. (S12), (S29) and (S35). These expectation values do not take into account the removal of

patients from a screening cohort when adenoma become malignant. For a full stochastic treatment the expectation values of Dewanji et al. [3] should be applied.

For $K = 0$ Heidenreich [5] has shown that the hazard $h(t)$ propagates to

$$h(t + \mathit{\Delta} t) = E[C(t)|y > 0]\, h_c(\mathit{\Delta} t) + h(\mathit{\Delta} t), \tag{S47}$$

which is expressed as the sum of the hazard from already existing initiated cells at time t and the hazard from freshly generated cells in time interval $\mathit{\Delta} t$. The hazard $h_c(\mathit{\Delta} t)$ pertains to a single cell present at time t which becomes malignant after a delay $\mathit{\Delta} t$. Since these rare transforming events occur independently, the total hazard from existing cells is obtained multiplying $E[C(t)|y > 0]$ to $h_c(\mathit{\Delta} t)$. For $K = 1, 2$ similar relations hold. Note, that $h_c(0) = \nu$.

Transformation probability

With Eq. (S46) the hazard function at age $t + \Delta t$ for an adenoma at age t with $Y(t)$ cells is given by

$$h_Y(t+\Delta t) = Y(t)\nu(t)T(t,t+\Delta t) \quad \text{with time progation factor} \tag{S48}$$
$$T(t,t+\Delta t) = \frac{E_c(t+\Delta t)\nu(t+\Delta t) - E_c(\Delta t)\nu(\Delta t)}{E_c(t)\nu(t)} \quad \text{and}$$
$$E_c(t) = E[C(t)|y>0]$$

defined analogously to $h_c(\Delta t)$ in Eq. (S47).

In our simplified cancer induction model the adenoma is transformed into a clinically relevant tumor with age-dependent rate $\nu(t)$ of Eq. (2). Age dependence must be taken into account since ν increases substantially with older age.

The probability of having an adenoma transformed at time $t + \Delta t$ is then given by

$$p_Y(t+\Delta t) = \int_t^{t+\Delta t} h_Y(s)S_Y(s)ds \quad \text{with survival function} \tag{S49}$$
$$\ln(S_Y(t)) = -\int_0^t h_Y(s)ds$$

We omit competing risks which can be easily accounted for by replacing $S_Y(s) \to S_Y(s)S_c(s)$ in the integrand. By approximating integration with summation over M_t time intervals of width $\Delta s = \Delta t/M_t$ we obtain

$$p_Y(t+\Delta t) = S_Y(t) \sum_{i=1}^{M_t} h_Y(t+i\Delta s/2) \exp\left(-\sum_{j=1}^{i} h_Y(t+j\Delta s/2)\Delta s\right) \Delta s. \tag{S50}$$

We can discard $S_Y(t)$ when we assume that an adenoma is always present at age t .

Quantifying screening efficiency

If optimal screening at age t removes all adenoma above the detection limit y_0, the cell-weighted probabilities for remaining cells in adenoma below the detection limit are given by

$$1 \times P[Y(t) = 1|y > 0] = \frac{\Theta(1,t)}{E[N(t)|y > 0]} \tag{S51}$$
$$\dots$$
$$y_0 \times P[Y(t) = y_0|y > 0] = \frac{\Theta(y_0,t)}{E[N(t)|y > 0]}.$$

Hence, the expectation value for the number of remaining cells per adenoma larger than y_0 at age t can be written as

$$\begin{aligned} R[Y(t)|y > y_0] &= E[Y(t)|y > 0] - \sum_{y=1}^{y_0} \frac{y\Theta(y,t)}{E[N(t)|y > 0]} \\ &= E[Y(t)|y > 0] - E[Y(t)|y > y_0]\,\frac{E[N(t)|y > y_0]}{E[N(t)|y > 0]}. \end{aligned} \tag{S52}$$

The corresponding expectation value for the number of remaining adenoma is expressed as

$$R[N(t)|y > y_0] = E[N(t)|y > 0] - E[N(t)|y > y_0]. \tag{S53}$$

The expected number of remaining cells per individual becomes

$$R[C(t)|y > y_0] = R[N(t)|y > y_0]R[Y(t)|y > y_0]. \tag{S54}$$

In reality the optimal screening efficiency for a given detection limit y_0 is never achieved. Adenoma removal depends on external factors such as the

experience of the colonoscopist. These observations suggest a simple model assumption for screening success. It is assumed that screening reduces the number of adenoma and also changes their size distribution.

To quantify screening efficiency we define two efficiency factors $0 \leq r_N^{eff}, r_Y^{eff} \leq 1$ pertaining to the removal of adenoma (r_N^{eff}) and to the reduction of initiated cells per adenoma (r_N^{eff}). Optimal screening efficiency means the complete removal of all detectable adenoma and is defined by $r_N^{eff} = r_Y^{eff} = 1$.

Finally, the expected effective number of remaining cells per patient becomes

$$R_{eff}[C(t)|y > y_0] = (E[N(t)|y > 0] - r_N^{eff}\, E[N(t)|y > y_0]) \times (E[Y(t)|y > 0] - r_Y^{eff}\, E[Y(t)|y > y_0]\, \frac{r_N^{eff}\, E[N(t)|y > y_0]}{E[N(t)|y > 0]}). \tag{S55}$$

Hazard ratio for risk decrease

With Eq. (S55) the hazard ratio for risk decrease in a screening cohort is written as

$$\mathrm{HR}_{eff}(t + \Delta t) = \frac{R_{eff}[C(t)|y > y_0]h_c(\Delta t) + h(\Delta t)}{h(t + \Delta t)}. \tag{S56}$$

Eq. (S56) also constitutes an analytical expression for the hazard ratio of interval cancer which has been measured by Corley et al. [6]. Interval cancers are diagnosed in patients after they had a colonoscopy.

Since ν cancels out, for $\Delta t = 0$ the hazard ratio

$$\begin{aligned}\mathrm{RR}_{eff}(t) &= \frac{R_{eff}[C(t)|y > y_0]}{E[C(t)|y < y_0]} \\ &= \left(\frac{r_N^{eff} E_N^{y_0}(t)}{E_N^0(t)} - 1\right)\left(\frac{r_N^{eff} E_N^{y_0}(t)}{E_N^0(t)} \frac{r_Y^{eff} E_Y^{y_0}(t)}{E_Y^0(t)} - 1\right)\end{aligned} \tag{S57}$$

pertains to the share of remaining cells at age of examination t as a fraction of all initiated cells in a patient. The abbreviation $E_N^{y_0}(t) = E[N(t)|y > y_0]$ applies to the remaining expectation values, correspondingly.

For $\Delta t < 10$ yr after a screening examination the hazard $h(\Delta t)$ for *de novo* created tumors can be neglected in Eq. (S56). We use this observation to equate

$$\mathrm{RR}_{eff}(t) \simeq \mathrm{HR}_{eff}(t + \Delta t) \tag{S58}$$

which enables a direct comparison of the reported hazard ratios of Corley et al. [6] with estimates from the present study.

S2 Supplementary Figures

Number of patients and measured adenoma detection rate

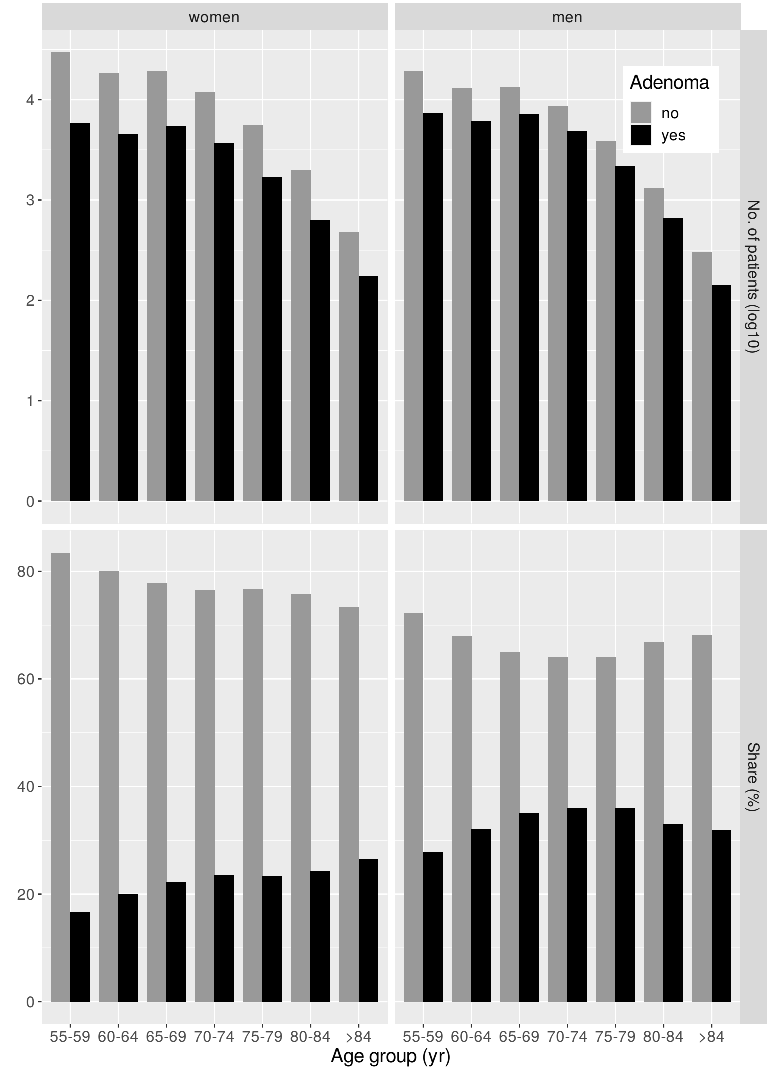

Fig. A Number of patients (A) and measured adenoma detection rate (B) in 5 yr age groups for all adenoma shapes combined.

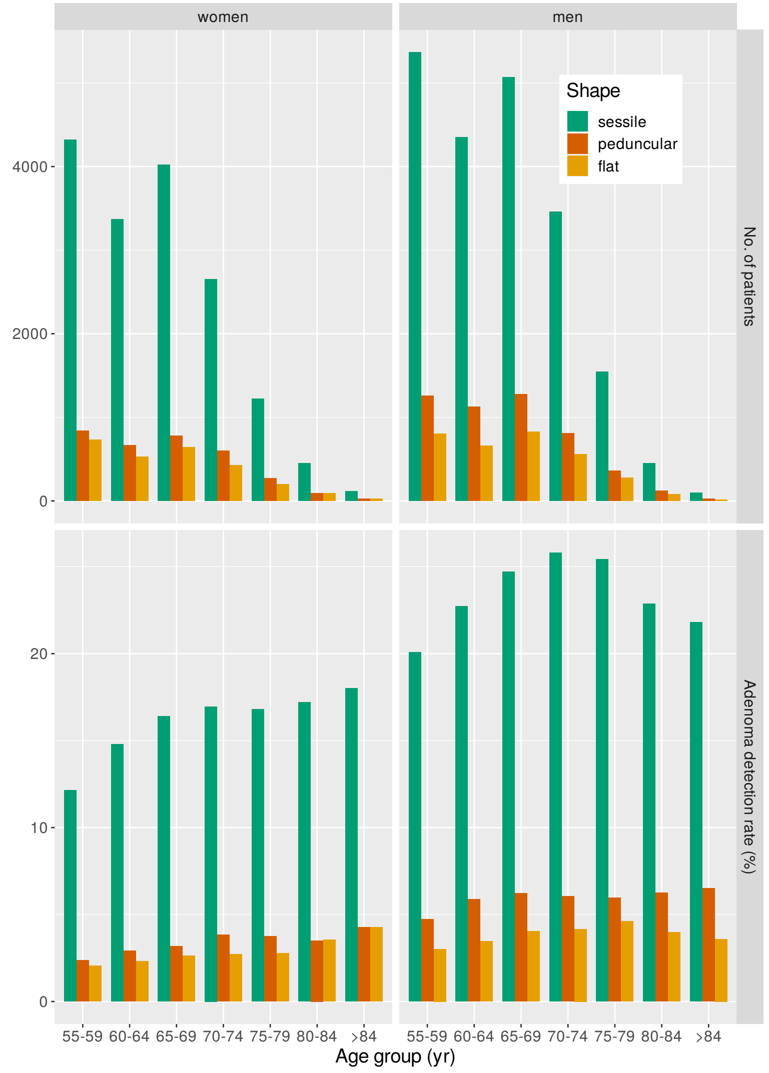

Fig. B Number of patients (A) and measured adenoma detection rate (B) in 5 yr age groups for shapes sessile, peduncular and flat; if more than one adenoma was reported, the shape of the most advanced adenoma was counted.

Age dependence of parameters in growth models

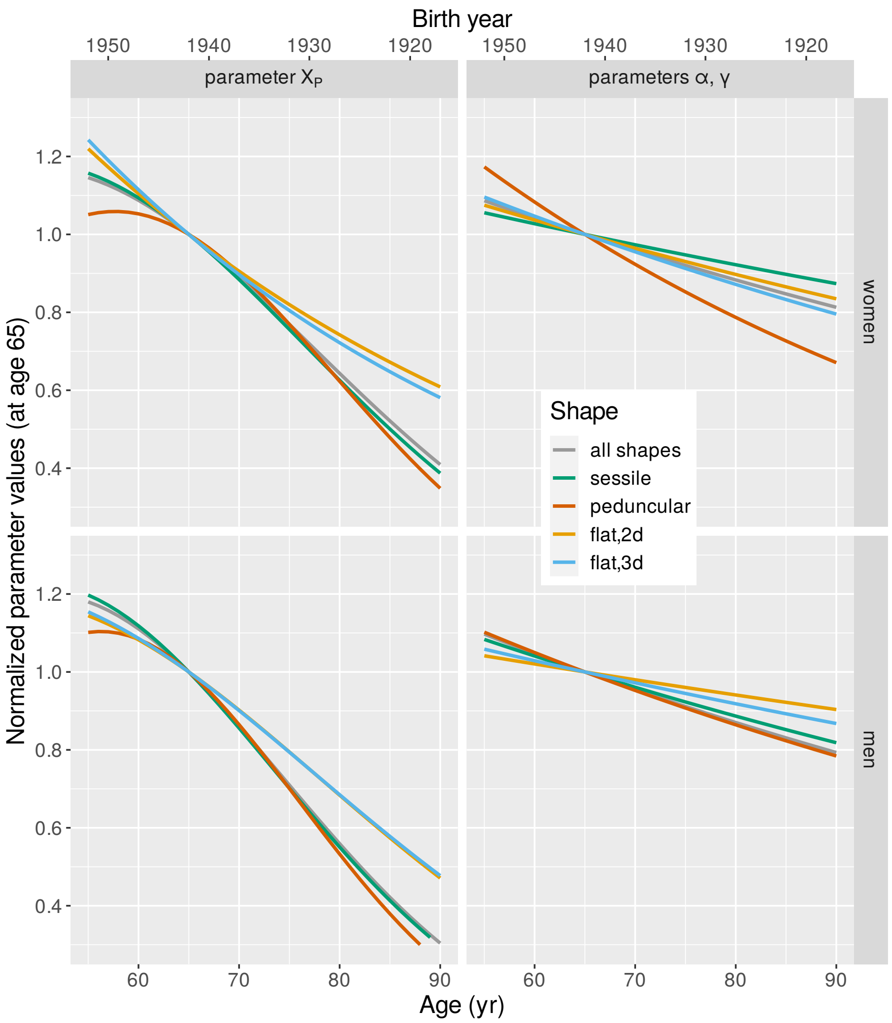

Fig. C Age dependence of Poisson strength X_P, cell division rate α and clonal growth rate γ defined in Eq. (3), age dependence of initiation parameter $\rho(t) = \mu_2/\alpha(t)$ is not shown, parameter estimates and 95% CI are given in Tables G - K, parameter values are normalized to age 65, birth years on the top x-axis pertain to 2007 - age.

Age dependence of the transformation rate in cancer risk models

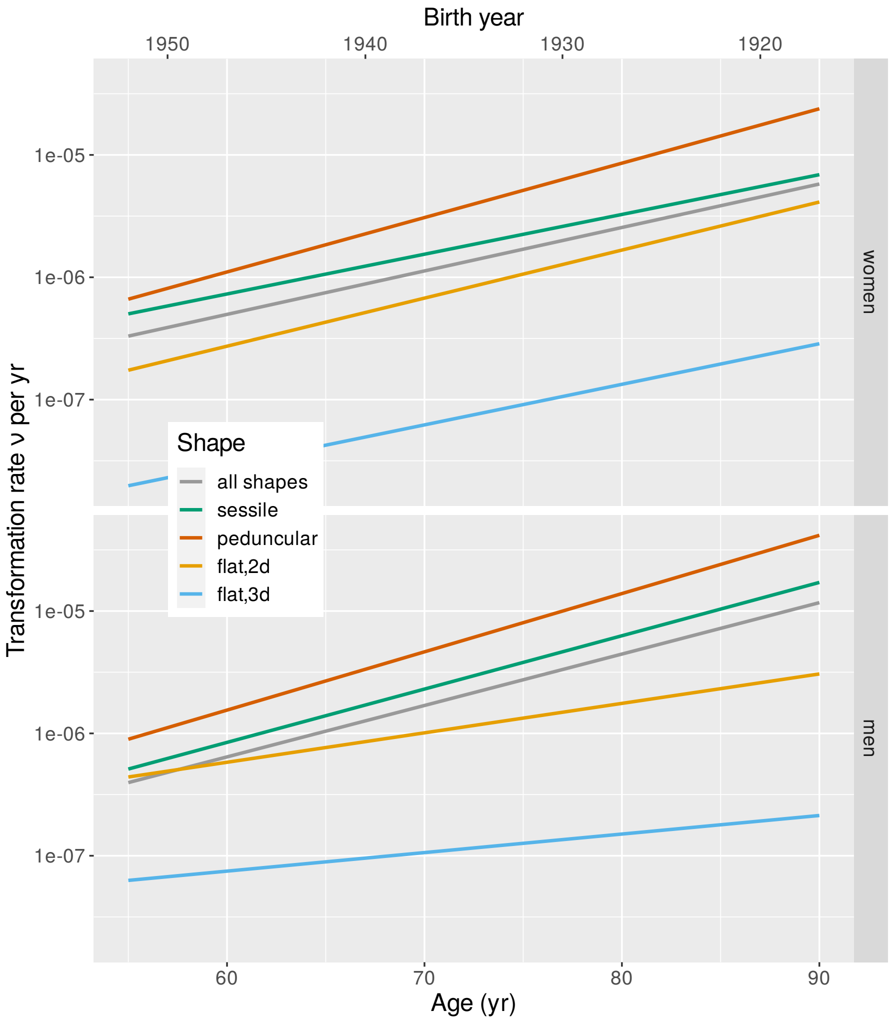

Fig. D Age dependence of the transformation rate ν defined in Eq (2), parameter estimates and 95% CI are given in Table L, birth years on the top x-axis pertain to 2007 - age.

Adenoma count distribution for all shapes combined

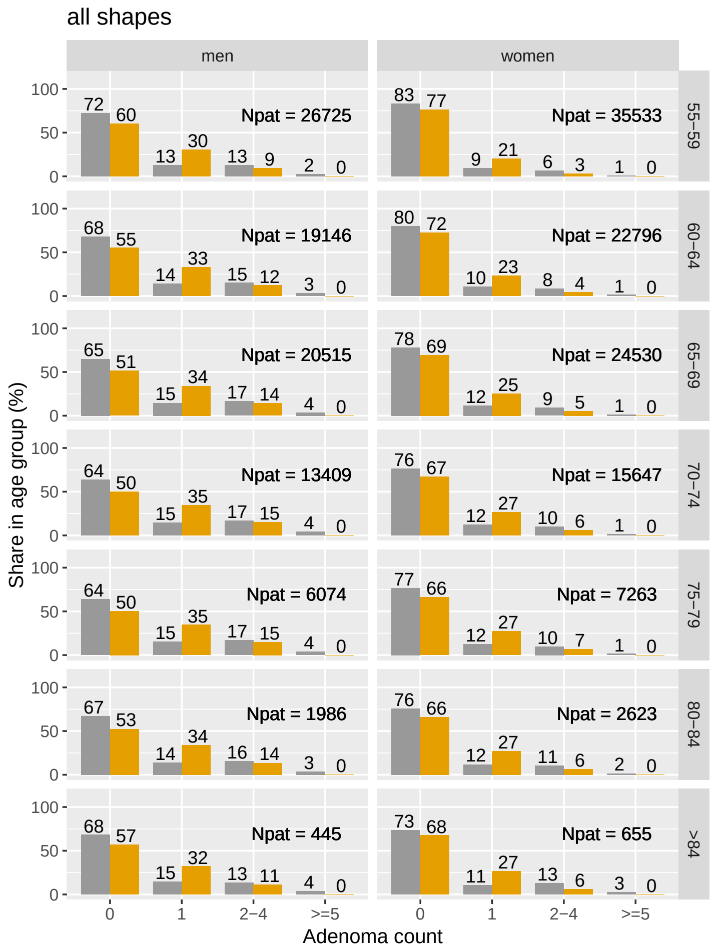

Fig. E Adenoma counts of all shapes combined for men and women in 5 yr age groups from screening (left bars) and from model expectations (right bars), adenoma detection rate (ADR) is given by 1 - Share(count = 0), N_{pat} denotes the number of patients in each panel.

Adenoma size distribution for all shapes combined

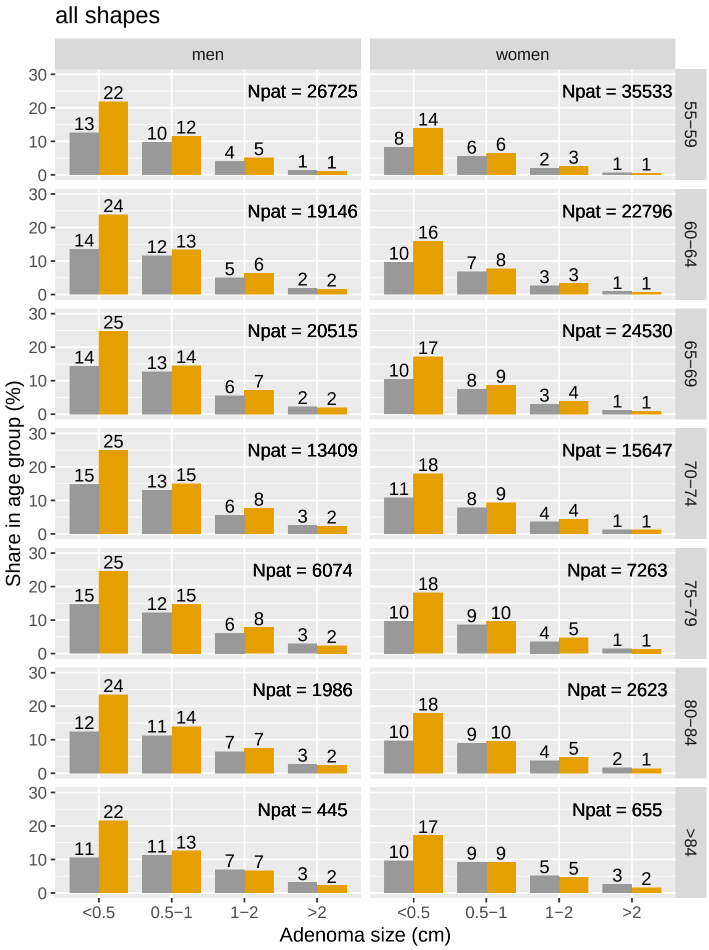

Fig. F Adenoma size distribution of all shapes combined for men and women in 5 yr age groups from screening (left bars) and from model expectations (right bars). If more than one adenoma is detected, the size category is derived from the size of the most advanced adenoma according to Table A, N_{pat} denotes the number of patients in each panel.

S3 Supplementary Tables

Table A Probabilities $P(\text{size} = sc, \text{counts} = cc)$ of finding a patient with most advanced adenoma in size category sc and count category cc, for comparison with recorded adenoma the probabilities $P(\text{size} = sc) = \sum_{cc} P(\text{size} = sc, \text{counts} = cc)$ in the last column have been used.

	adenoma counts per patient			counts sum
size (cm)	1	2-4	≥ 5	$P(\text{size} = sc)$
< 0.5	$d_1 p_1$	$\sum_{n=2}^{4} d_n p_1^n$	$\sum_{n=5}^{\infty} d_n p_1^n$	$e^{\lambda(p_1-1)} - d_0$
0.5 - 1	$d_1 p_2$	$\sum_{n=2}^{4} d_n \times \left[(p_1+p_2)^n - p_1^n\right]$	$\sum_{n=5}^{\infty} d_n \times \left[(p_1+p_2)^n - p_1^n\right]$	$e^{\lambda(p_1+p_2-1)} - e^{\lambda(p_1-1)}$
1 - 2	$d_1 p_3$	$\sum_{n=2}^{4} d_n \times [(1-p_4)^n - (p_1+p_2)^n]$	$\sum_{n=5}^{\infty} d_n \times [(1-p_4)^n - (p_1+p_2)^n]$	$e^{-\lambda p_4} - e^{\lambda(p_1+p_2-1)}$
≥ 2	$d_1 p_4$	$\sum_{n=2}^{4} d_n [1-(1-p_4)^n]$	$\sum_{n=5}^{\infty} d_n [1-(1-p_4)^n]$	$1 - e^{-\lambda p_4}$
size sum	d_1	$\sum_{n=2}^{4} d_n$	$\sum_{n=5}^{\infty} d_n$	$1 - d_0 =$ [a]ADR

[a] adenoma detection rate

Breakdown of screening data in age groups

Table B Number of female and male patients N_{pat} and number of detected adenoma N_{ad} in 5 yr age groups.

age group (yr)	N_{pat} all	N_{pat} [a]adeno.	N_{pat} [b]sessile	[b]peduncular	[b]flat	N_{ad} [c]all shapes
			women			
55 - 59	35533	5889	4320	839	730	9385
60 - 64	22796	4564	3369	666	529	7588
65 - 69	24530	5444	4021	778	645	9066
70 - 74	15647	3681	2654	601	426	6136
75 - 79	7263	1695	1221	273	201	2786
80 - 84	2623	636	451	92	93	1093
> 84	655	174	118	28	28	331
total	109047	22083	16154	3277	2652	36385
			men			
55 - 59	26725	7436	5368	1261	807	13391
60 - 64	19146	6141	4352	1126	663	11432
65 - 69	20515	7173	5068	1278	827	13643
70 - 74	13409	4829	3459	811	559	9332
75 - 79	6074	2188	1545	362	281	4113
80 - 84	1986	657	454	124	79	1242
> 84	445	142	97	29	16	269
total	88300	28566	20343	4991	3232	53422

[a] with reported adenoma, [b] most advanced adenoma, [c] for count categories $2-4$, ≥ 5 counts were set to 2 and 5 as most probable values

Colorectal cancers detected from 2006 to 2008

Table C Number of female and male patients N_{pat}, number of detected colorectal cancers N_{can} and crude incidence rate cr for 10^4 persons per year in 5 yr age groups.

age	all	all shapes		sessile		peduncular		flat	
group (yr)	N_{pat}	N_{can}	cr	N_{can}	cr	N_{can}	cr	N_{can}	cr
				women					
55 - 59	22478	11	0.36	8	0.27	1	0.033	2	0.066
60 - 64	14710	19	0.91	13	0.62	4	0.19	2	0.096
65 - 69	16592	25	1.9	17	1.3	7	0.53	1	0.076
70 - 74	9618	17	2.9	10	1.7	5	0.84	2	0.34
75 - 79	4523	12	5.3	6	2.6	2	0.88	4	1.8
80 - 84	1652	3	4.8	2	3.2	1	1.6	0	0
> 84	411	2	18	2	18	0	0	0	0
total	70084	89	1.2	58	0.79	20	0.27	11	0.15
				men					
55 - 59	17449	30	1.1	18	0.72	8	0.30	4	0.15
60 - 64	13822	39	2.2	22	1.2	11	0.59	6	0.32
65 - 69	15190	53	4.8	34	3.1	14	0.96	5	0.44
70 - 74	8574	42	8.3	24	4.7	12	2.0	6	1.2
75 - 79	3929	28	15	19	10	6	2.9	3	1.7
80 - 84	1123	11	25	5	11	5	10	1	2.5
> 84	253	4	47	3	35	1	14	0	0
total	60340	207	3.4	125	2.0	57	0.90	25	0.39

Flow of control in regression analysis

Table D Flow of control in regression analysis.

level	models	fit approach	purpose
I	$K = 0, 1, 2$	no trends in attained age, all parameters free, fits for 2d + 3d	rough estimation of parameter values
II	$K = 0, 1, 2$	attained age trends in X_P, α, γ, ρ with α_0, ρ_0 fixed	identification of growth dimensionality
		fits for 2d + 3d	selection of preferred models
[a]III	preferred	parameter estimation as in level II, variation of y_0	estimation of detection limit
[b,c] sCRM	preferred	level III parameters fixed	cancer risk estimation
	+ TR	parameters of transformation rate (TR) free	

[a] estimated quantities: distribution and mean of adenoma size, distribution and mean of adenoma number (APC), adenoma detection rate (ADR), extinction probability in Eq. (S45) by simulation

[b] simple Cancer Risk Model as product of adenoma growth model (level III) and transformation rate (TR)

[c] estimated quantities: cancer hazard, transformation probability in Eq. (S50), hazard ratio of interval cancer RR_{eff} in Eqs. (5, S57)

Goodness-of-fit for models of levels I and II

Table E Overall goodness-of-fit measured by AIC for all shapes combined, sex-specific models for $K = 0$, $K = 1$ and $K = 2$ with constant parameters (level I) and age dependences (3) in parameters X_{PI}, α and γ (level II), cell growth was assumed in either 2d or 3d dimensions according to Eq. (1).

		all shapes	
Level I	Model	2d	3d
women	K0	206885	209974
	K1	[a]**204341**	205002
	K2	204528	205345
men	K0	233860	237840
	K1	[a]**230949**	231796
	K2	231642	231774
Level II	Model	2d	3d
women	K0	206543	209480
	K1	203949	204401
	K2	[a]**203340**	204169
men	K0	233484	237361
	K1	230060	230599
	K2	[a]**229131**	229318

[a] lowest AIC

Table F Overall goodness-of-fit measured by AIC shapes sessile, peduncular and flat separately, sex-specific models for $K = 0$, $K = 1$ and $K = 2$ with constant parameters (level I) and age dependences (3) in parameters X_{PI}, α and γ (level II), cell growth was assumed in either 2d or 3d dimensions according to Eq. (1).

		sessile		peduncular		flat	
Level I	Model	2d	3d	2d	3d	2d	3d
women	K0	161820	164916	52379	52630	46018	46282
	K1	[a]**159916**	160661	[a]**52231**	52334	[a]**45723**	45768
	K2	160017	160400	52303	52356	45753	45757
men	K0	185746	189792	75325	75746	55786	56176
	K1	[a]**183447**	184453	[a]**75209**	75432	[a]**55373**	55434
	K2	183828	184155	75400	75610	55409	55406
Level II	Model	2d	3d	2d	3d	2d	3d
women	K0	161692	164701	52251	52473	45976	46219
	K1	159618	160268	52186	52255	45700	45725
	K2	[a]**159032**	159565	[a]**52160**	52242	45656	[a]**45639**
men	K0	185561	189549	75218	75620	55744	56107
	K1	182734	183544	75113	75267	55334	55384
	K2	[a]**181934**	182486	[a]**75103**	75258	55259	[a]**55253**

[a] lowest AIC

Goodness-of-fit and parameter estimates for models of level III

Table G Adenoma with all shapes combined: goodness-of-fit and parameter estimates for the preferred model $K = 2$ (level III), 2d growth and detection limit 30 cells (0.19 cm), age dependences in parameters X_{PI}, α and γ from Eq. (3), p-values < 0.001.

parameter	unit	[a]MLE	[b]SE	[c]2.5% CI	[c]97.5% CI
women		AIC 202532			
$X_{PI} = N\mu_0\mu_1$	yr^{-2}	0.0179	0.0001	0.0176	0.0182
α	yr^{-1}	10.0		fixed	
γ	yr^{-1}	0.0681	0.0006	0.0670	0.0683
ρ	-	4.40e-3		fixed	
b_{x_1}	yr^{-1}	-0.199	0.008	-0.214	-0.186
b_{x_2}	yr^{-1}	-0.0632	0.085	-0.0788	-0.0463
b_a	yr^{-1}	-0.0829	0.0084	-0.0985	-0.0662
men		AIC 227989			
X_{PI}	yr^{-2}	0.0514	0.0003	0.0508	0.0521
α	yr^{-1}	11.7		fixed	
γ	yr^{-1}	0.0673	0.0005	0.0664	0.0683
ρ	-	2.65e-3		fixed	
b_{x_1}	yr^{-1}	-0.254	0.007	-0.268	-0.241
b_{x_2}	yr^{-1}	-0.0888	0.0076	-0.1036	-0.0739
b_a	yr^{-1}	-0.0927	0.0075	-0.1073	-0.0785

[a] Maximum Likelihood Estimate [b] Standard Error

[c] Confidence Interval from likelihood profile

Table H Sessile adenoma: goodness-of-fit and parameter estimates for the preferred model $K = 2$ (level III), 2d growth and detection limit 30 cells (0.19 cm), age dependences in parameters X_{PI}, α and γ from Eq. (3) , p-values < 0.001.

parameter	unit	[a]MLE	[b]SE	[c]2.5% CI	[c]97.5% CI
women		AIC 158180			
$X_{PI} = N\mu_0\mu_1$	yr^{-2}	0.0738	0.0007	0.0725	0.0752
α	yr^{-1}	7.1		fixed	
γ	yr^{-1}	0.0580	0.0006	0.0567	0.0593
ρ	-	9.49e-4		fixed	
b_{x_1}	yr^{-1}	-0.213	0.010	-0.231	-0.194
b_{x_2}	yr^{-1}	-0.0667	0.010	-0.0868	-0.0470
b_a	yr^{-1}	-0.0540	0.0102	-0.0739	-0.0339
men		AIC 180665			
X_{PI}	yr^{-2}	0.149	0.0012	0.147	0.152
α	yr^{-1}	8.9		fixed	
γ	yr^{-1}	0.0554	0.0006	0.0543	0.0565
ρ	-	7.59e-4		fixed	
b_{x_1}	yr^{-1}	-0.267	0.008	-0.284	-0.251
b_{x_2}	yr^{-1}	-0.0872	0.0092	-0.1052	-0.0617
b_a	yr^{-1}	-0.0802	0.0094	-0.0987	-0.0336

[a] Maximum Likelihood Estimate [b] Standard Error

[c] Confidence Interval from likelihood profile

Table I Peduncular adenoma: goodness-of-fit and parameter estimates for the preferred model $K = 2$ (level III), 2d growth and detection limit 40 cells (0.45 cm), age dependences in parameters X_{PI}, α and γ from Eq. (3), p-values < 0.001 unless indicated otherwise.

parameter	unit	[a]MLE	[b]SE	[c]2.5% CI	[c]97.5% CI
women		AIC 52133			
$X_{PI} = N\mu_0\mu_1$	yr^{-2}	0.00399	8.3e-5	0.00380	0.0418
α	yr^{-1}	10.0		fixed	
γ	yr^{-1}	0.0370	0.0015	0.0341	0.0390
ρ	-	4.40e-3		fixed	
b_{x_1}	yr^{-1}	-0.156	0.022	-0.193	-0.120
b_{x_2}	yr^{-1}	-0.106	0.022	-0.145	-0.080
b_a	yr^{-1}	-0.160	0.030	-0.214	-0.105
men		AIC 75103			
X_{PI}	yr^{-2}	0.0609	0.0009	0.0590	0.0628
α	yr^{-1}	28.6		fixed	
γ	yr^{-1}	-0.0093	0.0017	-0.0126	-0.0061
ρ	-	5.15e-4		fixed	
b_{x_1}	yr^{-1}	-0.226	0.033	-0.287	-0.160
b_{x_2}	yr^{-1}	-0.129	0.018	-0.165	-0.094
b_a	yr^{-1}	[d]-0.0972	0.0484	-0.1885	0.0016

[a] Maximum Likelihood Estimate [b] Standard Error

[c] Confidence Interval from likelihood profile

[d] p = 0.045

Table J Flat adenoma: goodness-of-fit and parameter estimates for the model $K = 2$ (level III), 2d growth and detection limit 30 cells (0.19 cm), age dependences in parameters X_{PI}, α and γ from Eq. (3), p-values < 0.001 unless indicated otherwise.

parameter	unit	[a]MLE	[b]SE	[c]2.5% CI	[c]97.5% CI
women		AIC 45590			
$X_{PI} = N\mu_0\mu_1$	yr^{-2}	0.00777	1.4e-4	0.00747	0.00801
α	yr^{-1}	17.0		fixed	
γ	yr^{-1}	0.0749	0.0019	0.0714	0.0788
ρ	-	1.01e-3		fixed	
b_{x_1}	yr^{-1}	-0.199	0.021	-0.237	-0.159
b_{x_2}	yr^{-1}		p > 0.05		
b_a	yr^{-1}	[d]-0.0723	0.0240	-0.1173	-0.0251
men		AIC 55167			
X_{PI}	yr^{-2}	0.0305	0.0006	0.0296	0.0314
α	yr^{-1}	13.8		fixed	
γ	yr^{-1}	0.0772	0.0016	0.0743	0.0803
ρ	-	4.76e-4		fixed	
b_{x_1}	yr^{-1}	-0.1823	0.033	-0.287	-0.160
b_{x_2}	yr^{-1}	-0.0475	0.018	-0.165	-0.094
b_a	yr^{-1}	[e]-0.0406	0.0204	-0.0801	-0.0008

[a] Maximum Likelihood Estimate [b] Standard Error

[c] Confidence Interval from likelihood profile

[d] p = 2.6e-3

[e] p = 4.7e-2

Table K Flat adenoma: goodness-of-fit and parameter estimates for the preferred model $K = 2$ (level III), 3d growth and detection limit 30 cells (0.21 cm), age dependences in parameters X_{PI}, α and γ from Eq. (3), p-values < 0.001 unless indicated otherwise.

parameter	unit	[a]MLE	[b]SE	[c]2.5% CI	[c]97.5% CI
women		AIC 45584			
$X_{PI} = N\mu_0\mu_1$	yr^{-2}	0.0124	0.0002	0.0119	0.0128
α	yr^{-1}	11.8		fixed	
γ	yr^{-1}	0.151	0.002	0.147	0.156
ρ	-	4.76e-4		fixed	
b_{x_1}	yr^{-1}	-0.217	0.020	-0.255	-0.178
b_{x_2}	yr^{-1}		$p > 0.05$		
b_a	yr^{-1}	-0.0915	0.0175	-0.1255	-0.0573
men		AIC 55171			
X_{PI}	yr^{-2}	0.00135	1.9e-5	0.00131	0.00139
α	yr^{-1}	25.9		fixed	
γ	yr^{-1}	0.130	0.002	0.126	0.133
ρ	-	9.41e-4		fixed	
b_{x_1}	yr^{-1}	-0.187	0.020	-0.222	-0.153
b_{x_2}	yr^{-1}	[d]-0.0436	0.021	-0.0839	-0.0079
b_a	yr^{-1}	[d]-0.0568	0.0165	-0.0894	-0.0237

[a] Maximum Likelihood Estimate [b] Standard Error

[c] Confidence Interval from likelihood profile

[d] $p = 3.4e\text{-}2$

Parameter estimates for the transformation rate

Table L Parameter estimates for the transformation rate $\nu(t) = \nu_0 \exp\left[(b_n\,(t-65)/10\right]$ in the simple cancer risk model, p-values < 0.001 unless indicated otherwise.

Sex	[a]MLE	[b]SE	[c]2.5%	[c]97.5%	[a]MLE	[b]SE	[c]2.5%	[c]97.5%
	ν_0 (10^{-6} yr^{-1})				b_n (yr^{-1})			
all shapes combined, 2d								
women	0.75	0.09	0.59	0.93	0.82	0.14	0.54	1.09
men	1.04	0.08	0.90	1.20	0.97	0.09	0.78	1.15
sessile, 2d								
women	1.06	0.15	0.80	1.37	0.75	0.18	0.40	1.09
men	1.39	0.14	1.15	1.68	1.00	0.12	0.77	1.24
peduncular, 2d								
women	1.84	0.47	1.09	2.86	1.02	0.29	0.44	1.59
men	2.68	0.39	1.99	3.51	1.10	0.18	0.75	1.44
flat, 2d								
women	0.43	0.16	0.20	0.77	[d]0.90	0.39	0.11	1.67
men	0.77	0.16	0.49	1.12	[e]0.55	0.27	0.003	1.08
flat, 3d								
women	0.042	0.015	0.020	0.076	[f]0.76	0.40	-0.05	1.54
men	0.089	0.019	0.057	0.130	[g]0.35	0.28	-0.21	0.88

[a] Maximum Likelihood Estimate [b] Standard Error

[c] Confidence Interval from likelihood profile

[d] p = 0.022, [e] p = 0.042, [f] p = 0.057, [g] p = 0.21

References

1. Jeon J, Meza R, Moolgavkar SH, and Luebeck EG. Evaluation of screening strategies for pre-malignant lesions using a biomathematical approach. Math Biosci 2008 May; 213 (1) :56–70. DOI: 10.1016/j.mbs.2008.02.006
2. Dewanji A, Luebeck EG, and Moolgavkar SH. A generalized Luria-Delbrück model. Math Biosci 2005 Oct; 197 (2) :140–52. DOI: 10.1016/j.mbs.2005.07.003
3. Dewanji A, Jeon J, Meza R, and Luebeck EG. Number and Size Distribution of Colorectal Adenomas under the Multistage Clonal Expansion Model of Cancer. PLoS Comput Biol 2011 Oct; 7 (10) :e1002213. DOI: 10.1371/journal.pcbi.1002213
4. Luebeck EG, Curtius K, Jeon J, and Hazelton WD. Impact of tumor progression on cancer incidence curves. Cancer Research 2013; 73 (3) :1086–96. DOI: 10.1158/0008-5472.CAN-12-2198
5. Heidenreich WF. Heterogeneity of cancer risk due to stochastic effects. Risk Anal 2005 Dec; 25 (6) :1589–94. DOI: 10.1111/j.1539-6924.2005.00685.x
6. Corley DA, Jensen CD, Marks AR, Zhao WK, Lee JK, Doubeni CA, Zauber AG, Boer J de, Fireman BH, Schottinger JE, Quinn VP, Ghai NR, Levin TR, and Quesenberry CP. Adenoma detection rate and risk of colorectal cancer and death. N Engl J Med 2014 Apr; 370 (14) :1298–306. DOI: 10.1056/NEJMoa1309086
